# Synthesis and Properties Evolution of a Family of Tiara-like Phenylethanethiolated Palladium Nanoclusters

**DOI:** 10.1038/srep16628

**Published:** 2015-11-16

**Authors:** Jishi Chen, Liren Liu, Linhong Weng, Yuejian Lin, Lingwen Liao, Chengming Wang, Jinlong Yang, Zhikun Wu

**Affiliations:** 1 Key Laboratory of Materials Physics, Anhui Key Laboratory of Nanomaterials and Nanotechnology, Institute of Solid State Physics, Chinese Academy of Sciences, Hefei 230031, China; 2 Department of Materials Science and Engineering, University of Science and Technology of China, Hefei, 230026, China; 3 Hefei National Laboratory for Physics Sciences at the Microscale, University of Science and Technology of China, Hefei, 230026, China; 4 Shanghai Key Laboratory of Molecular Catalysis and Innovative Materials, Department of Chemistry, Fudan University, Shanghai, 200433, China

## Abstract

Tiara-like thiolated group 10 transition metal (Ni, Pd, Pt) nanoclusters have attracted extensive interest due to their fundamental scientific significance and potential application in a number of fields. However, the properties (*e.g.* the absorption) evolution with the ring size’s increase was not investigated so far to our best knowledge, due to the challenge of obtaining a series of nanocluster analogues. Herein, we successfully synthesized, isolated and identified a family of [Pd(SC_2_H_4_Ph)_2_]_n_ nanoclusters (totally 17 novel clusters, n = 4–20). Their structures were determined to be tiara-like by single crystal X-ray crystallography together with theoretical calculation; their formation mechanism was proposed to be a substitution—polycondensation—ring-closure process based on experimental observations. All of these clusters are rather robust (anti-reductive and anti-oxidative) owing to their tiara-like structures with large HOMO-LUMO gaps. Finally, the optical and electrochemical evolution with the increase of ring size was investigated, and it is found that both optical and electrochemical gaps have a “turning point” at a size corresponding to n = 8 for [Pd(SR)_2_]_n_ nanoclusters.

Ligand-protected metal nanoclusters^1^ have attracted significant research interest in recent years because of their intriguing molecular-like properties^2^,^3^,^4^ and promising applications in a variety of fields such as biolabeling^5^, catalysis^6^, medicine^7^, photovoltics^8^, sensing^9^, renewable energy^10^, etc. Of the various metal nanoclusters, tiara-like thiolated group 10 transition metal (Ni, Pd and Pt) clusters (denoted as [M(SR)_2_]_n_) not only show interesting structures, but also exhibit intriguing properties such as superior nonlinear optical property^11^, photocatalytic hydrogen generation property^12^,^13^ and host-guest chemical property^11^,^14^,^15^,^16^. Their toroidal architectures are composed of a limited number of M(SR)_2_ units with two doubly bridged thiolate ligands between each adjacent pair.

After the first successful obtaining of tiara-like [Ni(SC_2_H_5_)_2_]_6_ through the reaction of organotin-sulfur compounds with nickel(II) chloride^17^, various methods such as the sand bath^18^ and the solvothermal method^19^ have been developed to synthesize double-crown [M(SR)_2_]_n_. However, at most two [M(SR)_2_]_n_ (*e.g.* [Pd(S-*n*Pr)_2_]_6_ and [Pd(S-*n*Pr)_2_]_8_) were jointly isolated^20^. There are currently no reports of a homologous series of thiolated metal clusters, which are highly desirable for materials science applications because they are produced through a one-pot method and some interferences can be avoided during the comparison of properties^21^ (*e.g.* introduced trace of impurities from various starting materials).

On the other hand, the size results are seriously unbalanced (see Table S1). Although tiara-like nickel complexes based on diverse thiolate ligands ([Ni(SR)_2_]_n_ with n = 4 ~ 6, 8 ~ 12^11^,^12^,^13^,^14^,^15^,^16^,^17^,^18^,^19^,^22^,^23^,^24^,^25^,^26^,^27^,^28^,^29^,^30^,^31^,^32^,^33^,^34^,^35^,^36^,^37^,^38^,^39^) have been prepared (Table S1), only two sizes of Pd clusters ([Pd(SR)_2_]_6_^18^,^20^,^40^,^41^,^42^,^43^,^44^ and [Pd(SR)_2_]_8_^20^,^45^) and one Pt cluster ([Pt(SCH_2_COOMe)_2_]_8_^45^) have been reported. Additional questions that naturally arise include: Are there any other sized Pd (Pt) clusters beside the reported [Pd(SR)_2_]_6_^18^,^20^,^40^,^41^,^42^,^43^,^44^ and [Pd(SR)_2_]_8_^20^,^45^ ([Pt(SCH_2_COOMe)_2_]_8_^45^)? Are there any other tiara-like [M(SR)_2_]_n_ with n value bigger than 12^11^, *etc.*? To unravel these intriguing issues, specifically, to investigate the property evolution with the increase of ring size, phenylethanethiolated Pd clusters were chosen for the study because Pd is less noble than Pt and phenylethanethiol was a good ligand for metal nanoclusters. Herein, we successfully synthesized, isolated and identified a continuous series of [Pd(SC_2_H_4_Ph)_2_]_n_ clusters (4 ≤ n ≤ 20; Pd_n_ for short) with tiara-like structure. Furthermore, we suggest a “substitution—polycondensation—ring-closure” formation mechanism for the tiara structures, and demonstrate the optical and electrochemical evolution of the homologous series.

## Results and Discussion

### Synthesis and separation

**Figure 1:**
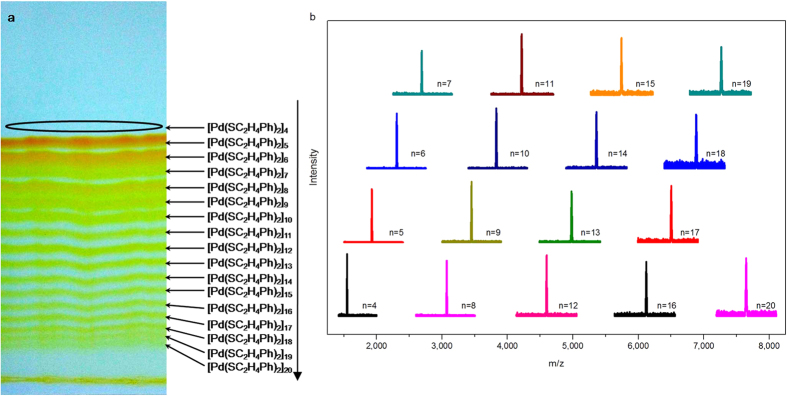
PTLC photograph (**a**) and MALDI-TOF-MS spectra (**b**) of [Pd(SC_2_H_4_Ph)_2_]_n_ (4 ≤ n ≤ 20).

The homologous series of phenylethanethiolated Pd clusters were synthesized based on a previous method^14^. In a typical synthesis, a deoxygenized acetonitrile solution containing Pd(NO_3_)_2_•2H_2_O was mixed with 2 equivalents of 2-phenylethanethiol and 2 equivalents of triethylamine (Note: argon atmosphere is employed to avoid any possible interference from air, but it is not essential for this reaction). After continuously stirred for 5 hrs, the reaction mixture was extracted with CH_2_Cl_2_. Then, we thoroughly isolated the components in the crude product via preparative thin layer chromatography (PTLC), which was common in the purification of organic compounds but is rarely used for the isolation of tiara-like metal clusters. Surprisingly and interestingly, the extracted product in our protocol contains at least 17 components as shown by PTLC (see Fig. 1). Of note, at the top of the PTLC plate, there is another band almost indiscernible (marked with an oval, Fig. 1a).

The full separation of the continuous series of [Pd(SC_2_H_4_Ph)_2_]_n_ (4 ≤ n ≤ 20) is challenging, and the identification of all the 17 compounds is even more challenging. No distinct signal from M/Z of 1000 to 10000 in the MALDI-TOF-MS spectrum was found in either positive or negative ionization mode even if an excellent matrix—trans-2-[3-(4-tert-Butylphenyl)-2-methyl-2-propenyldidene] malononitrile (DCTB) was used^46^,^47^. Other matrixes including sinapinic acid (SA), 2,5-dihydroxybenzoic acid (DHB), alpha-cyano-4-hydroxycinnamic acid (CHCA), and cesium acetate do not work either. Fortunately, the addition of NaOOCCF_3_ assisted in the ionization of the clusters as shown in Fig. 1b and S1 probably due to the inclusion of Na^+^ in the cave of double-crown structure^17^. The major peaks are readily assigned to [[Pd(SC_2_H_4_Ph)_2_]_n_ + Na]^+^ (4 ≤ n ≤ 20) based on the M/Z value in tandem with the isotopic patterns in the mass spectra (380 × n + 23; 380 is the M/Z value of Pd(SC_2_H_4_Ph)_2_ unit, and 23 is the M/Z value of the Na cation). Only one size of thiolated Pd cluster is found in the mass spectrum of every band in PTLC plate (see Fig. 1b and S1), indicating that all the seventeen compounds are well isolated and purified by PTLC.

Of note, Pd_5_ and Pd_6_ are the two main components (see Fig. 1a). These were obtained at 5%, and 4% yield, respectively. The yield of Pd_n_ is less than that of Pd_n+1_ (n are odd numbers between 7 and 19), and the yields of Pd_n_ generally decrease as values of n increase (n > 4). Thus, it is difficult to obtain Pd_n_ (n > 10) considering their yields are very low (less than 1%). Furthermore, the products distribution is found to be immune to changes in experimental parameters (for examples, changing the feeding ratio of Pd(NO_3_)_2_:PhC_2_H_4_SH from 1:2 to 1:6; turning the solvent acetonitrile to n-propanol, replacing the base triethylamine with NaBH_4_, withdrawing the argon atomosphere, etc.). These products can be dried and fully re-dispersed in dichloromethane, chloroform, dichloroethane, toluene, ethylacetate and DMF easily, but they can not dissolve in alcohol, acetonitrile, n-hexane and petroleum ether. All of the Pd_n_ (4 ≤ n ≤ 20) are novel—even the other cluster sizes except [Pd(SR)_2_]_6_ and [Pd(SR)_2_]_8_ have not been reported, to the best of our knowledge. Although [Pd(SR)_2_]_3_^43^ and [Ni(SR)_2_]_3_^48^ have been theoretically predicted, the practical existence of these species is still problematic because they are less obviously stable than the larger ones^43^,^48^, and the smallest cluster obtained in this work is Pd_4_. [Ni(μ-S*t*Bu)(μ-etet)]_12_ was the biggest tiara-like [M(SR)_2_]_n_ nanocluster synthesized and identified previously, herein we shatter the record by providing larger Pd_20_.

### Structure of [Pd(SC_2_H_4_Ph)_2_]_n_ (4 ≤ n ≤ 20)

**Figure 2:**
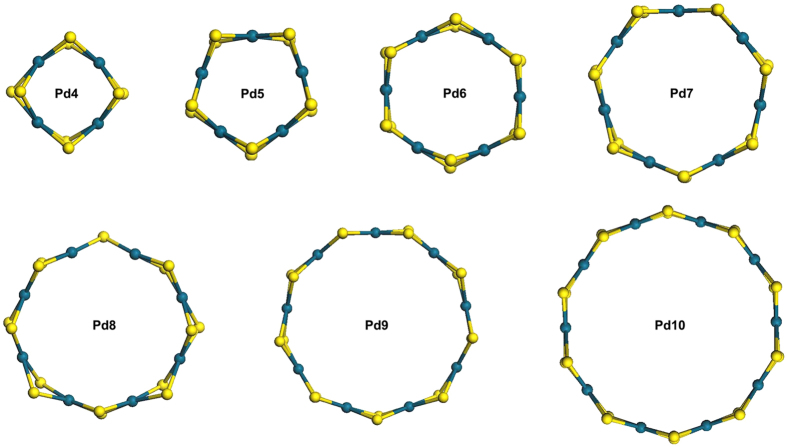
Optimized structure of [Pd(SC_2_H_4_Ph)_2_]_n_ (carbon and hydrogen atoms were omitted for clarity). Blue, Pd; yellow, S.

The following facts exclude that [Pd(SC_2_H_4_Ph)_2_]_n_ (4 ≤ n ≤ 20) adopt chain structure. First, the Pd/S atomic ratio of all the series is exactly 1:2. In chained Pd-thiolate polymer, 2n-2 bridging ligands and 4 terminal ligands are needed to keep the plane dsp^2^ orbital hybridization of Pd atom, thereby the Pd/S atomic ratio is n:(2n+2). Second, short-chained Pd-thiolate polymers in solution can continue to grow longer until the growth process is inhibited by some interferences (*e.g.* solubility). Third, the regular distribution of variously sized Pd clusters (for instance, the even-odd law) implies that they probably adopt similar structures as ones reported previously (see Table S1)^11^,^12^,^13^,^14^,^15^,^16^,^17^,^18^,^19^,^20^,^22^,^23^,^24^,^25^,^26^,^27^,^28^,^29^,^30^,^31^,^32^,^33^,^34^,^35^,^36^,^37^,^38^,^39^,^40^,^41^,^42^,^43^,^44^,^45^. Single crystal X-ray crystallography of [Pd(SC_2_H_4_Ph)_2_]_6_ demonstrate that six palladium atoms arranged in an approximately hexagonal ring, with six bridging sulfur atoms above and six below the plane (see Fig. S2). Theoretical calculations using density functional theory (DFT) also confirm that [Pd(SR)_2_]_n_ (4 ≤ n ≤ 10) adopt tiara structures (see Fig. 2 and S3).

### Synthesis mechanism

An intriguing issue pertains to the mechanism of formation in the continuous series of thiolated Pd clusters. There are two existing mechanisms for the formation of tiara-like [M(SR)_2_]_n_: one is termed as “template mechanical assistance”^14^,^16^, and the other is “addition polymerization of monomer”^45^. However, the two existing mechanisms are not well applied to this case. The “template mechanical assistance” is unsuitable because it is difficult to find the template of all 17 compounds and explain why a series of Pd_n_ (4 ≤ n ≤ 20) is formed from a single template. The “addition polymerization of monomer” is also inconsistent with our finding that NO_3_^−^ and H_2_O exist in the initial Pd-SR complex. Alternatively, we propose a formation mechanism named “substitution—polycondensation—ring-closure”.

**Figure 3:**
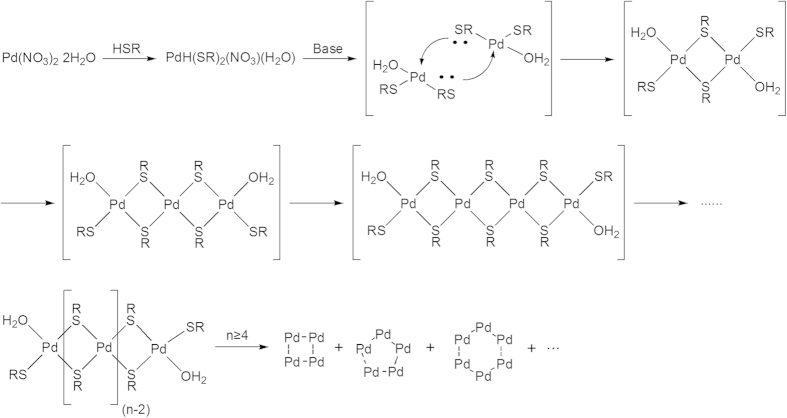
Flow chart of the synthesis of [Pd(SC_2_H_4_Ph)_2_]_n_ from the Pd(II) salt.

The entire process could be divided into three steps: first, palladium nitrate dihydrate reacts with 2 equivalents of thiol and immediately yields a yellow precipitate (see Fig. 3 and Eq. 1). The binding energy of Pd3d in the precipitate is 337.6 eV (Fig. S4). This indicates that Pd^2+^ is not reduced or oxidized and remains in its dsp^2^ orbital hybridization plane after reaction with phenylethanethiol. The peak at 1380 cm^−1^ in the IR spectrum (Fig. S5) indicates the existence of NO_3_^−^ in the precipitation. The absorption at ~3400 cm^−1^ in the IR spectrum suggests that one hydrated water may be still there. Thus, it is possible that two phenylethanethiols replace one NO_3_^−^ and one hydrated water molecule. As a result, the formula of the precipitate is PdH(SC_2_H_4_Ph)_2_(NO_3_)(H_2_O)—the acidity of the precipitate indicates phenylethanethiol may not be completely deprotonated (Fig. 3 and Eq. 1). This is supported by elemental analysis of sulfur (calculated: 13.95%; experimental: 13.80%).

Second, linear condensation of monomer (PdH(SC_2_H_4_Ph)_2_(NO_3_)(H_2_O) (the precipitate of the first step) occurs after base triggering (herein triethylamine is employed as the base). The IR spectra suggest the absence of NO_3_^−^ and H_2_O in the Pd clusters. Thus, the NO_3_^−^ and the proton may be removed in the second step with assistance of triethylamine. Importantly, the fact that the reaction does not proceed smoothly in acidic or neutral media suggests removal of a proton and NO_3_^−^ in this step by the attack of the base (triethylamine). The linear condensation of two monomers leads to the forming of a dimer (Eq. 2), and the condensation of the dimer with a monomer leads to the forming of trimer (Eq. 3). The condensation of two dimmers (Eq. 4) or one trimer with one monomer leads to tetramer, and so on. This process is comparable to the polycondensation of polymers.

Third, the resulting chains close into rings head to tail by detracting the hydrated water molecules (Eq. 5 and Fig. 3). The resulting double-crown structures do not contain hydrated water any longer, which is confirmed by the IR spectra (see Fig. S5). The composition was further supported by elemental analysis of sulfur (calculated: 16.84%; experimental: 16.91%). For the closure of odd Pd clusters, the modification of the configuration was needed before the ring-closure to avoid spatial hindrance. This is why the yields of odd Pd_n_ cluster are lower than those of the adjacent even Pd_n+1_ clusters.

In brief, the formation of Pd_n_ clusters is a substitution—polycondensation—ring-closure process based on the data. This is consistent with yield of every component in the product.


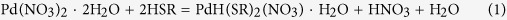



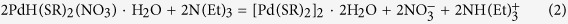



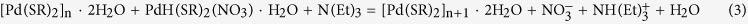



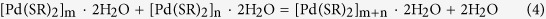



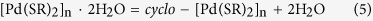


### Evolution of optical and electrochemical properties

**Figure 4:**
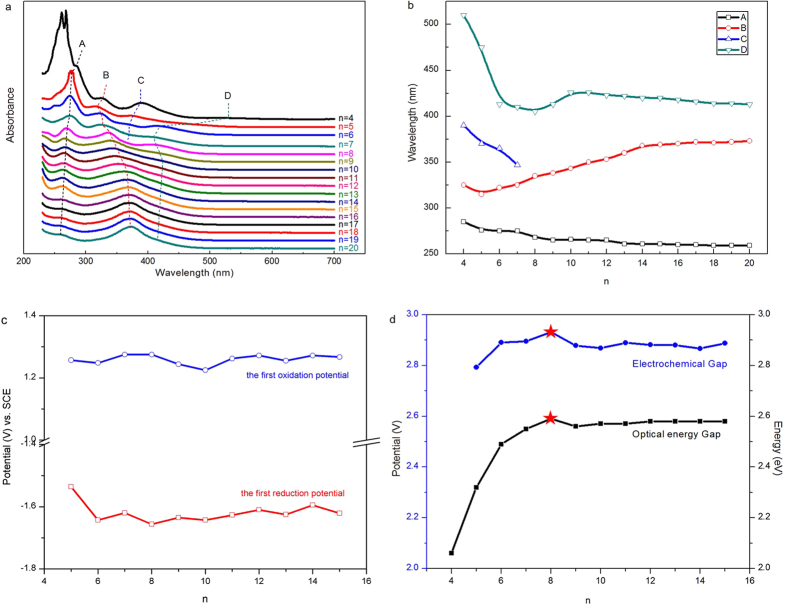
UV/Vis absorption spectra and differential pulse voltammetry (DPV) potential of Pd_n_ (n = 4 ~ 20) clusters. (**a**) UV/Vis absorption spectra of Pd_n_ (n = 4 ~ 20) clusters. (**b**) The UV/Vis absoption evolution of Pd_n_ (n = 4 ~ 20) clusters. (**c**) The first oxidation (reduction) potential evolution of Pd_n_ (n = 5 ~ 15) clusters. (**d**) The electrochemical and optic energy band gaps of Pd_n_ (n = 4 ~ 15) clusters.

To systematically compare the property evolutions of the full series of Pd_n_ clusters is meaningful because they have similar tiara-like structures, the same composition, and the same synthesis history. First, the UV/Vis absorption spectra were recorded for comparison. The optical evolution from Pd_4_ to Pd_20_ is interesting. The absorption spectra are similar, but the shifts in the absorption peaks are dramatic for small Pd_n_ (n is less than 9; see Fig. 4a,b). However, the shifts become static and almost linear when the n in Pd_n_ is larger than 8. Generally speaking, the peaks at 285 and 510 nm in the spectrum of Pd_4_ blueshifts, while the peak at 325 nm in the spectrum of Pd_4_ redshifts as the size of the Pd clusters increases. The peak at 390 nm in the spectrum of Pd_4_ blueshifts to 340 nm in the spectrum of Pd_7_, but it is indiscernible in the spectra of larger Pd_n_ clusters (8 ≤ n ≤ 20)—this may indicate some size effects of the tiara-like structure phenylethanethiolated Pd clusters: Strong quantum confinement of electrons might occur in the smaller Pd_n_ (n < 8) clusters, which results in discrete molecule-like electronic structure, and the orbit energy gap decreases with the size decrease. As a result, the absorption wavelength blueshifts from 390 nm to 340 nm accompanying with the size increase from Pd_4_ to Pd_7_.

The first reduction potentials of the phenylethanethiolated Pd_n_ (n > 6) clusters fluctuate at −1.62 V (−1.54 V for Pd_5_, see Fig. 4, S7 and Table S2). This is comparable to that of the previous tiara-like Ni_6_(SC_2_H_4_Ph)_12_^24^ and indicates that the Pd clusters is also anti-reductive, which is confirmed by the reduction experiments (see Fig. S8). Surprisingly, the first oxidation potential of all investigated phenylethanethiolated Pd clusters are almost consistent, and they are remarkably higher than that of Ni_6_(SC_2_H_4_Ph)_12_^24^ (1.26 vs 0.65 V), indicating that the phenylethanethiolated Pd clusters are very stable to oxidation, which is indeed consistent with the H_2_O_2_ oxidation experiments (see Fig. S9). The gaps between the first reduction potentials and the first oxidation potentials of phenylethanethiolated Pd clusters are summarized in Fig. 4d, which is also in agreement with the optical energy gap after considering the charge energy (0.29 V)^49^. The large energy gaps indicate that these clusters are rather robust, which was confirmed by additional experiments: Pd_6_ is stable in ambient environment for over 90 days and at 80 °C for over 24 hrs demonstrated by UV/Vis absorption spectra (see Fig. S10,11). A thermogravimetric analysis (TGA) of Pd_6_ is shown in Fig. S12, which indicates that Pd_6_ is stable until ~200 °C and discomposes completely at ~320 °C with a total weight loss of 62.9 wt% in the temperature range from 50 to 500 °C. However, there is some stability discrepancy at 80 °C between variously sized Pd clusters and it is shown that the stability decreases when the Pd clusters is larger than Pd_8_, see Fig. S11 for a comparison. Previous studies have shown that the energy gaps between the highest occupied molecular orbit and the lowest unoccupied molecular orbit (HOMO-LUMO gap) of some nanoscale semiconductors decrease with size increases^50^. However, in our case, the HOMO-LUMO gaps enlarge as the value of n in Pd_n_ increases from 4 to 8. When the value of n is larger than 8, the gaps slightly decrease and keep unchanged with the further increase of the n value, thus the “turning point” of HOMO-LUMO gaps and the largest gap lie at a size corresponding to n = 8 for Pd_n_ clusters.

In summary, we prepared, isolated and identified a family of novel[Pd(SC_2_H_4_Ph)_2_]_n_ clusters (totally 17 clusters, n = 4 ~ 20) with tiara-like structures, and propose their formation mechanism to be a substitution—polycondensation—ring-closure process. The optical and electrochemical properties evolutions from [Pd(SC_2_H_4_Ph)_2_]_4_ to [Pd(SC_2_H_4_Ph)_2_]_20_ were investigated, which indicate that the HOMO-LUMO gap “turning point” lie at a size corresponding to n = 8 for [Pd(SC_2_H_4_Ph)_2_]_n_ clusters: less than that size, the gap is size-dependent and increases with the size’s increase; whereas larger than the size, the gap are almost not influenced by size any more.

## Method

### Synthetic protocols

The reactions are conducted at room temperature under argon atmosphere. Briefly, Pd(NO_3_)_2_·2H_2_O (100 mg, 0.38 mmol) were dissolved in 6 ml acetonitrile in a 25 mL flask with vigorous stirring followed by PhC_2_H_4_SH (120 μL, 0.9 mmol) and triethylamine (120 μL, 0.87 mmol). After 5 hrs of stirring, yellow precipitates were collected, washed thoroughly by excess acetonitrile and methanol, and dried under reduced pressure. Mixed tiara-like clusters were extracted with CH_2_Cl_2_ and then separated and purified by PTLC (CH_2_Cl_2_: petroleum ether, 1/3 v:v). Needle-like [Pd(SC_2_H_4_Ph)_2_]_6_ single crystals were crystallized from the mixture of toluene and methanol at room temperature after 2 days.

### Characterization

The UV/Vis absorption spectra of Pd clusters (dissolved in CH_2_Cl_2_) were recorded in standard quartz cuvettes on a UV-2550 spectrophotometer (Shimadzu, Japan) at room temperature. Fourier transform infrared (FIIR) spectra were acquired on a Nicolet 8700 (Nicolet, America) spectrometer. MALDI-TOF-MS analyses were performed on an autoflex Speed TOF/TOF mass spectrometer (Bruker, Germany). Trans-2-[3-(4-tert-Butylphenyl)-2-methyl-2-propenyldidene] malononitrile (DCTB) was used as the matrix, and NaOOCCF_3_ was added to assist the ionization of clusters. X-ray Photoelectron Spectroscopy (XPS) measurements were conducted on an ESCALAB 250Xi XPS spectrometer (Thermo Scientific, America), using a monochromatized Al Kα source and equipped with an Ar+ion sputtering gun. All binding energies were calibrated using the C (1 s) carbon peak (284.8 eV). Elemental analyses were performed on vario EL III Elementar analyzer (S mode) (Elementar, Germany). Thermal gravimetric analysis (TGA) (~3 mg sample used) was conducted in a N_2_ atmosphere (flow rate ~ 50 mL/min) on a TG/DTA 6300 analyzer (Seiko Instruments, Inc), and the heating rate was 10 °C/min. The single crystal diffraction data of Pd_6_(SC_2_H_4_Ph)_12_ was collected on a Bruker APEXDUO X-ray Diffractometer (Bruker, Germany).

### Electrochemistry

A conventional three-electrode system was used for these experiments. A Pt disk electrode (1 mm diameter) was the working electrode (WE). Before use, the WE was polished on emery paper of decreasing grades followed by Al_2_O_3_ powders with sizes down to 0.05 μm. This was cleaned electrochemically with potential-cycling in 0.5 M H_2_SO_4_ solution, and the electrode was then rinsed thoroughly with ultrapure water (18.2 MΩ cm). An SCE (with saturated KCl solution) electrode and carbon rods served as the reference (RE) and counter electrode (CE), respectively. The electrode potentials were controlled with a potentiostat (Zahner, Germen). The Pd_n_ clusters were dissolved in 0.1 M Bu_4_NPF_6_ that was constantly purged with N_2_ (99.99% Nanjing Special Gas Corp.) during the experiments. All electrochemical experiments were carried out at room temperature (ca. 25 °C).

### Theoretical calculation

For details of theoretical calculation of structure and UV/Vis spectra, see the supplementary information.

## Additional Information

**How to cite this article**: Chen, J. *et al.* Synthesis and Properties Evolution of a Family of Tiara-like Phenylethanethiolated Palladium Nanoclusters. *Sci. Rep.* **5**, 16628; doi: 10.1038/srep16628 (2015).

## Supplementary Material

Supplementary Information
