# Supplementary material for: Synthesis and Properties Evolution of a Family of Tiara-like Phenylethanethiolated Palladium Nanoclusters
Source: Sci Rep. 2015 Nov 16;5:16628. doi: 10.1038/srep16628 (PMC4644969; doi:10.1038/srep16628)
Supplement: Supplementary Information [file srep16628-s1.pdf]

Supplementary Information

Synthesis and Properties Evolution of a Family of Tiara-like Phenylethanethiolated Palladium Nanoclusters

Jishi Chen,[1,2] Liren Liu,[3] Linhong Weng,[4] Yuejian Lin,[4] Lingwen Liao,[1] Chengming Wang,[3] Jin-long Yang,[3] and Zhikun Wu[1]

[1] Key Laboratory of Materials Physics, Anhui Key Laboratory of Nanomaterials and Nanostructures, Institute of Solid State Physics, Chinese Academy of Sciences, Hefei 230031, China

[2] Department of Materials Science and Engineering, University of Science and Technology of China, Hefei, 230026, China

[3] Hefei National Laboratory for Physics Sciences at the Microscale, University of Science and Technology of China, Hefei, 230026, China

[4] Shanghai Key Laboratory of Molecular Catalysis and Innovative Materials, Department of Chemistry, Fudan University, Shanghai, 200433, China

This supplementary information includes:

Chemicals

Theoretical calculation method

Figure S1 MALDI-MS of $[Pd(SC_2H_4Ph)_2]_n$ ($4 \leq n \leq 20$).

Figure S2 The single crystal structure of $[Pd(SC_2H_4Ph)_2]_6$.

Figure S3 Optimized molecular structure and optimized/experimental optical spectra of $[Pd(SCH_3)_2]_n$ ($4 \leq n \leq 10$).

Figure S4 XPS spectra.

Figure S5 FIIR absorption spectra.

Figure S6 UV/Vis absorption spectra of $[Pd(SC_2H_4Ph)_2]_n$ ($4 \leq n \leq 20$).

Figure S7 Differential pulse voltammetry (DPV) of $[Pd(SC_2H_4Ph)_2]_n$ ($5 \leq n \leq 15$).

Figure S8 Anti-reductive properties of $[Pd(SC_2H_4Ph)_2]_6$.

Figure S9 Anti-oxidative properties of $[Pd(SC_2H_4Ph)_2]_6$.

Figure S10 Long-term stability of $[Pd(SC_2H_4Ph)_2]_6$.

Figure S11 UV/Vis absorption spectra of toluene solutions of $[Pd(SC_2H_4Ph)_2]_n$ at 80 ^{o}C for different times under air atmosphere.

Figure S12 TGA of $[Pd(SC_2H_4Ph)_2]_6$.

Table S1 Summary of tiara-like $[M(SR)_2]_n$ (M=Ni, Pd, Pt) nanoclusters.

Table S2 Summary of differential pulse voltammetry (DPV) and UV/vis absorption results of $[Pd(SC_2H_4Ph)_2]_n$ ($5 \leq n \leq 15$).

Table S3. Crystal data and structure refinement for $[Pd(SC_2H_4Ph)_2]_6$.

Table S4. Atomic coordinates ($\times 10^4$) and equivalent isotropic displacement parameters ($Å^2 \times 10^3$) for $[Pd(SC_2H_4Ph)_2]_6$.

Table S5. Bond lengths [Å] and angles [°] for $[Pd(SC_2H_4Ph)_2]_6$.

Table S6. Anisotropic displacement parameters ($Å^2 \times 10^3$) for $[Pd(SC_2H_4Ph)_2]_6$.

Table S7. Hydrogen coordinates ($\times 10^4$) and isotropic displacement parameters ($Å^2 \times 10^3$) for $[Pd(SC_2H_4Ph)_2]_6$.

References.

Chemicals

2-phenylethanethiol (PhC_2H_4SH, 99%) was purchased from Sigma-Aldrich.

Palladium nitrate dihydrate ($Pd(NO_3)_2\cdot 2H_2O$, AR, 97.0%) was purchased from Shanghai chemical reagent co., ltd.

Acetonitrile (CH_3CN, AR grade, 99.0%), triethylamine ($N(Et)_3$, AR grade, 99.0%), methanol (CH_3OH, AR grade, 99.7%), dichloromethane (CH_2Cl_2, AR, 99.7%), n-hexane (C_6H_{14}, AR, 97.0%), toluene (C_7H_8, AR grade, 99.5%) were purchased from Sinopharm chemical reagent co., ltd.

All chemicals are commercially available and used as received except acetonitrile, through which Argon (99.99% Nanjing Special Gas Corp.) was bubbled. Nanopure water (resistivity 18.2 MΩ cm) was produced with a Barnstead NANO pure water system.

Theoretical calculation method

The geometry optimizations and the ultraviolet/visible (UV/Vis) absorption spectrum of $[Pd(SC_2H_4Ph)_2]_n$ ($4\leq n\leq 10$) nanoclusters were performed by using density functional theory (DFT) and time-dependentdensity functional theory (TDDFT) at B3LYP[S1-S4]/LANL2DZ[S5-S7]/6-31G*[S8,S9]level. The effective core potential (ECP) basis set LanL2DZ associated with the pseudopotential was used to describe the Pd atoms, whereas the basis sets of 6-31G* was used for C, S and H atoms. Gaussian band shape with a bandwidth of 0.4 eV was used to simulate the UV/vis spectrum. Due to computing capability, we used SCH_3 instead of SC_2H_4Ph for simplicity. All of the calculations in this work were carried out using the Gaussian09 program package[S10].

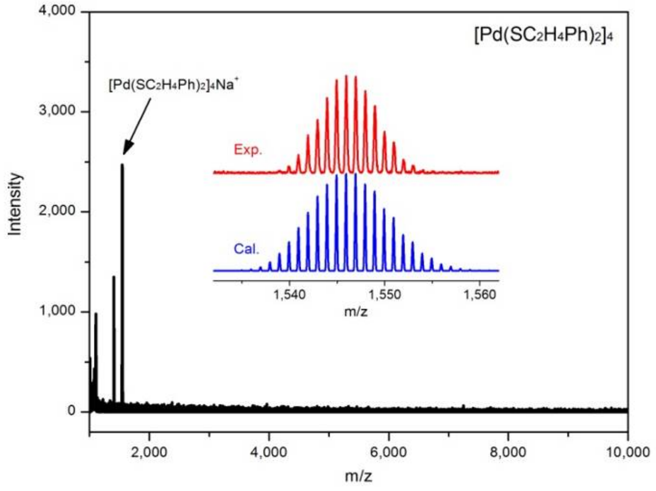
[Pd(SC2H4Ph)2]4
[Pd(SC2H4Ph)2]4Na+
Exp.
Cal.
Intensity
m/z

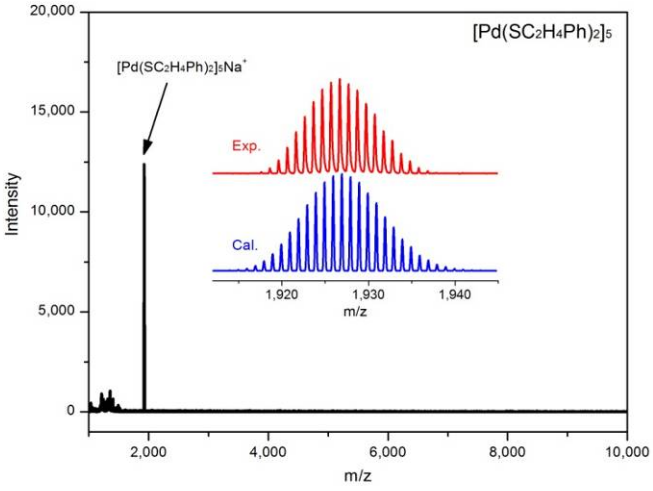
[Pd(SC2H4Ph)2]5
[Pd(SC2H4Ph)2]5Na+
Exp.
Cal.
Intensity
m/z

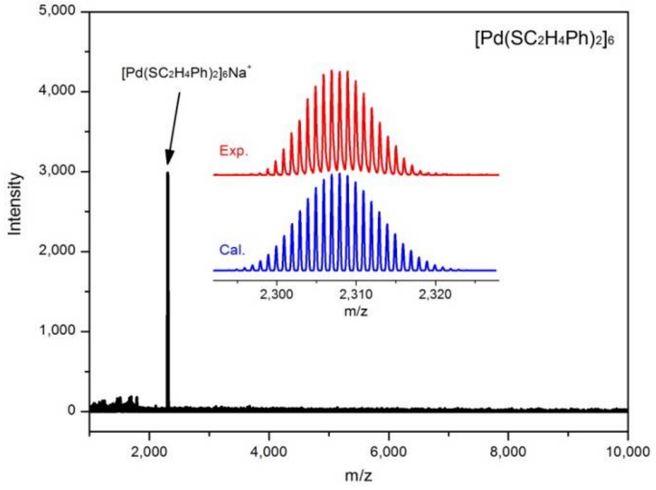
[Pd(SC2H4Ph)2]6
[Pd(SC2H4Ph)2]6Na+
Exp.
Cal.
Intensity
m/z

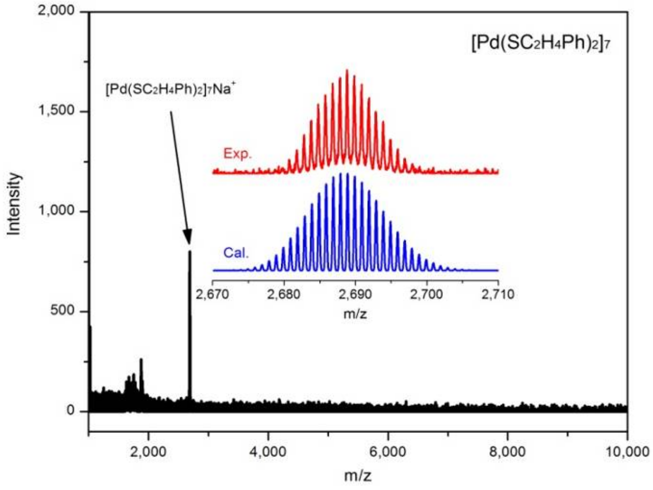
[Pd(SC2H4Ph)2]7
[Pd(SC2H4Ph)2]7Na+
Exp.
Cal.
Intensity
m/z

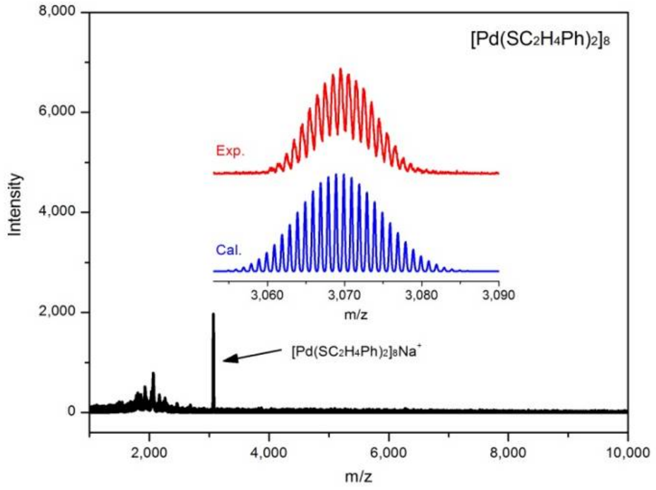
[Pd(SC2H4Ph)2]8
[Pd(SC2H4Ph)2]8Na+
Exp.
Cal.
Intensity
m/z

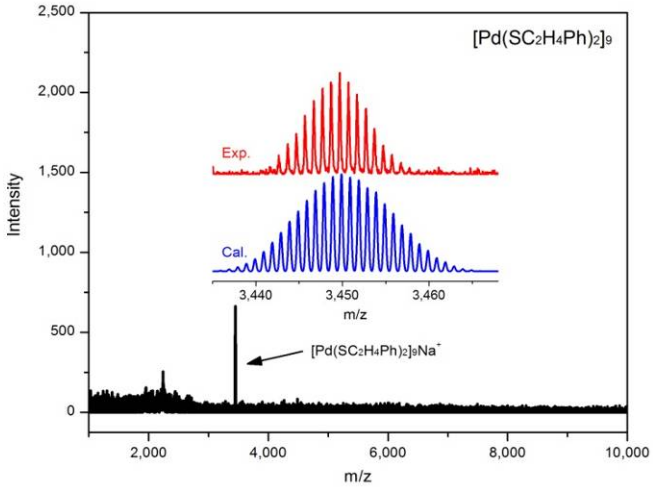
[Pd(SC2H4Ph)2]9
[Pd(SC2H4Ph)2]9Na+
Exp.
Cal.
Intensity
m/z

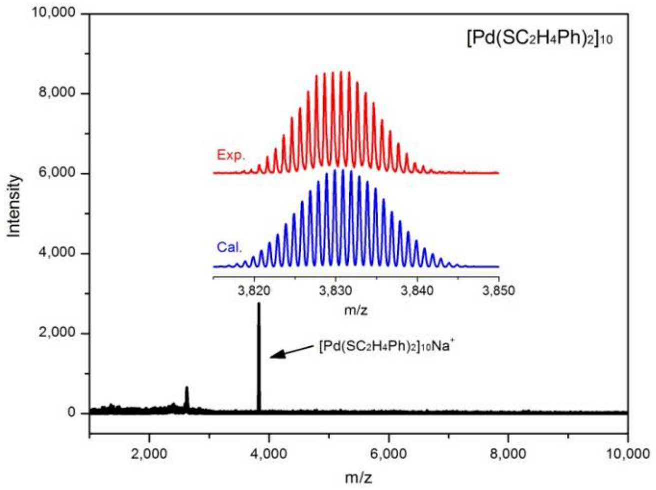
[Pd(SC2H4Ph)2]10
Exp.
Cal.
[Pd(SC2H4Ph)2]10Na+
Intensity
m/z

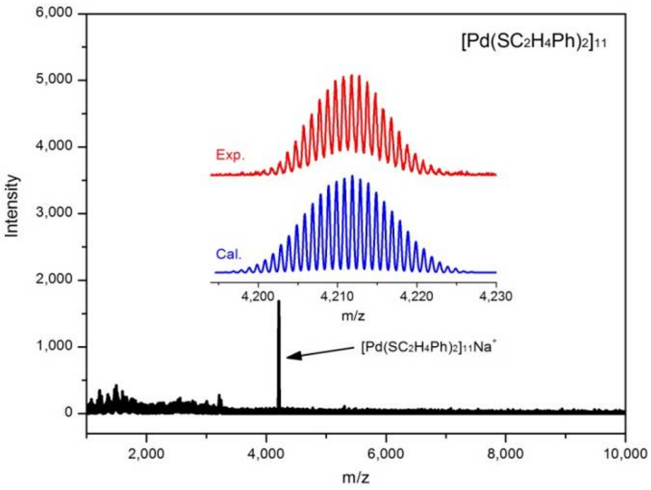
[Pd(SC2H4Ph)2]11
Exp.
Cal.
[Pd(SC2H4Ph)2]11Na+
Intensity
m/z

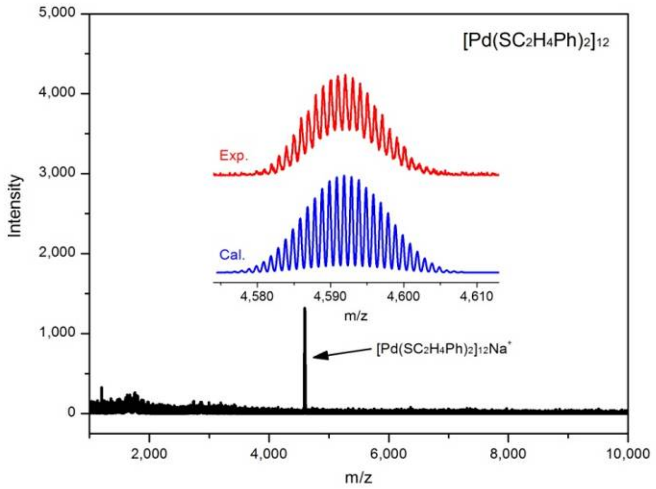
[Pd(SC2H4Ph)2]12
Exp.
Cal.
[Pd(SC2H4Ph)2]12Na+
Intensity
m/z

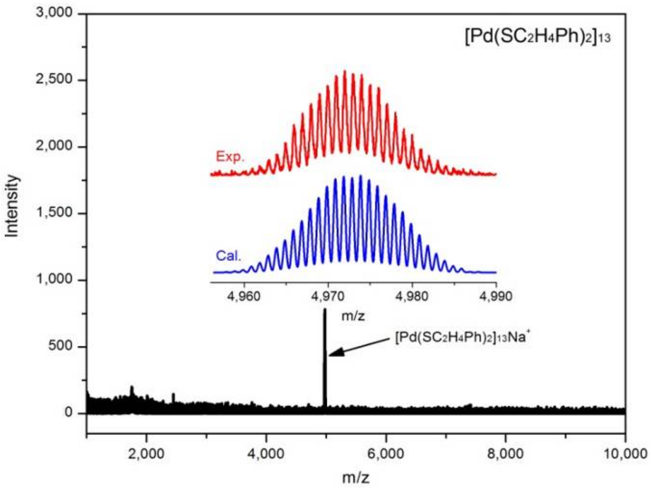
[Pd(SC2H4Ph)2]13
Exp.
Cal.
[Pd(SC2H4Ph)2]13Na+
Intensity
m/z

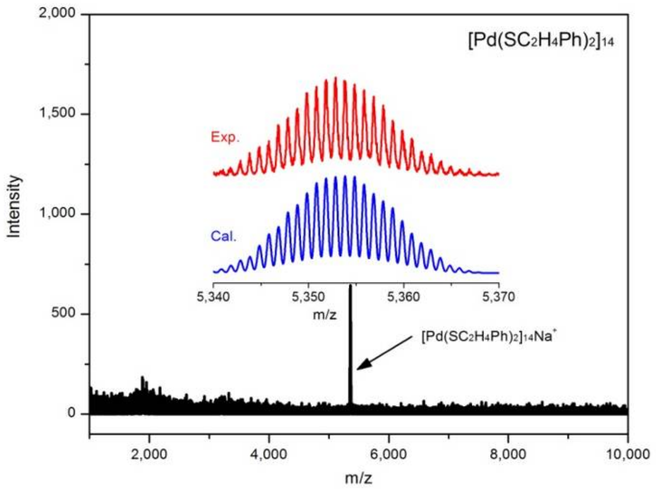
[Pd(SC2H4Ph)2]14
Exp.
Cal.
[Pd(SC2H4Ph)2]14Na+
Intensity
m/z

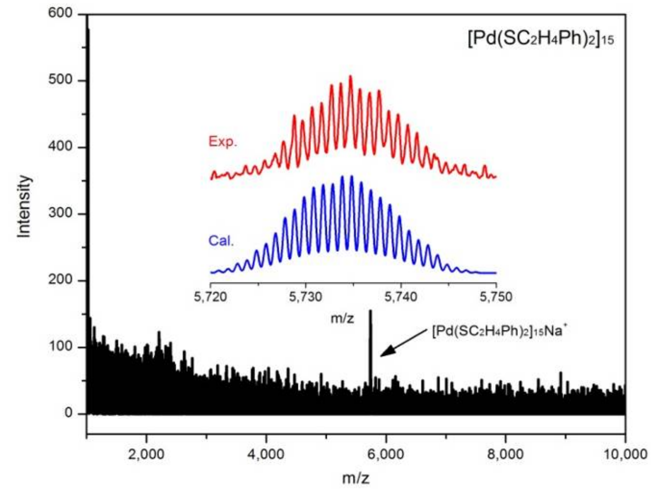
[Pd(SC2H4Ph)2]15
Exp.
Cal.
[Pd(SC2H4Ph)2]15Na+
Intensity
m/z

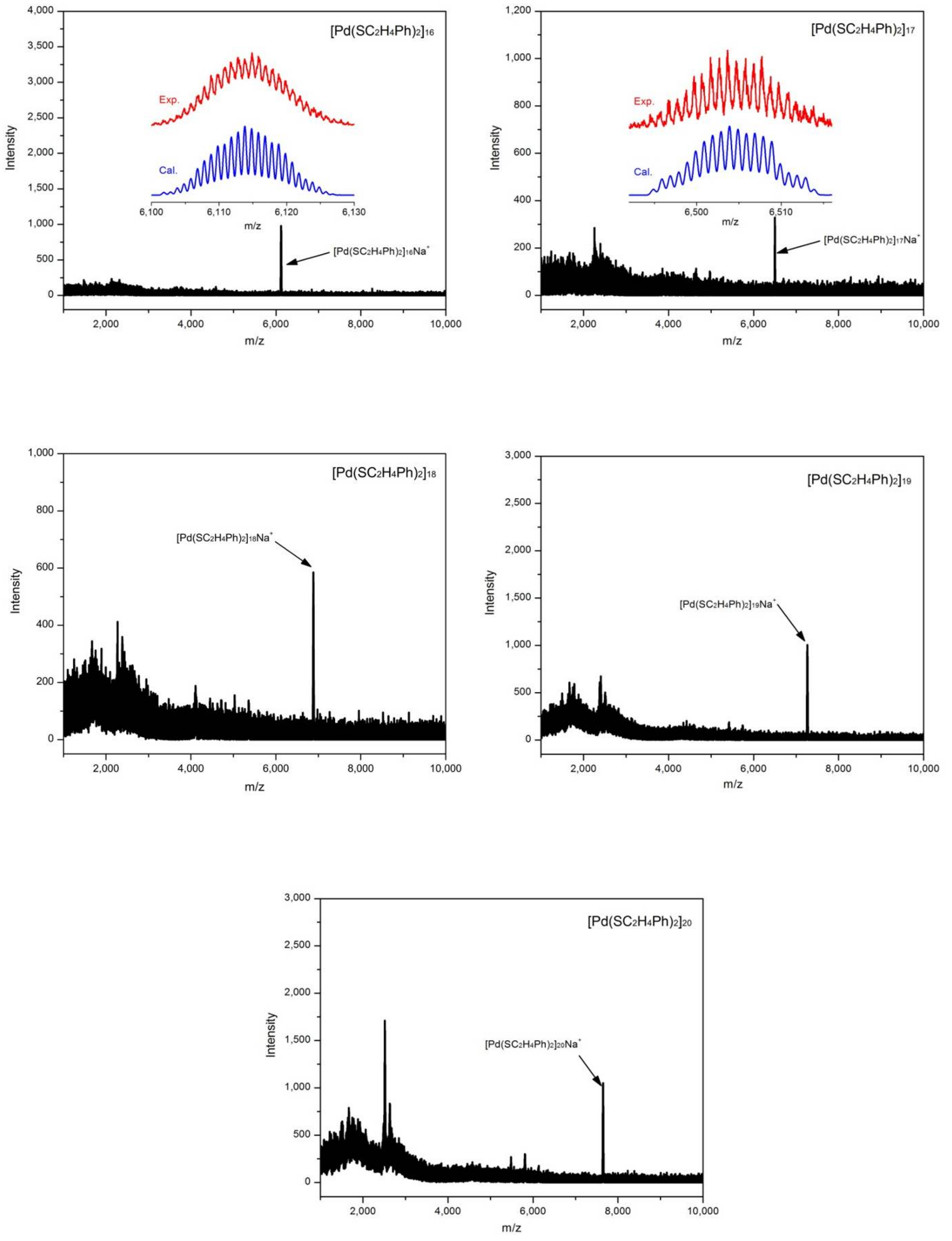

Figure S1 | MALDI-TOF-MS of $[Pd(SC_2H_4Ph)_2]_n$ (4≤n≤20). Inset is the isotopic patterns comparison (red: experimental; blue: calculated).

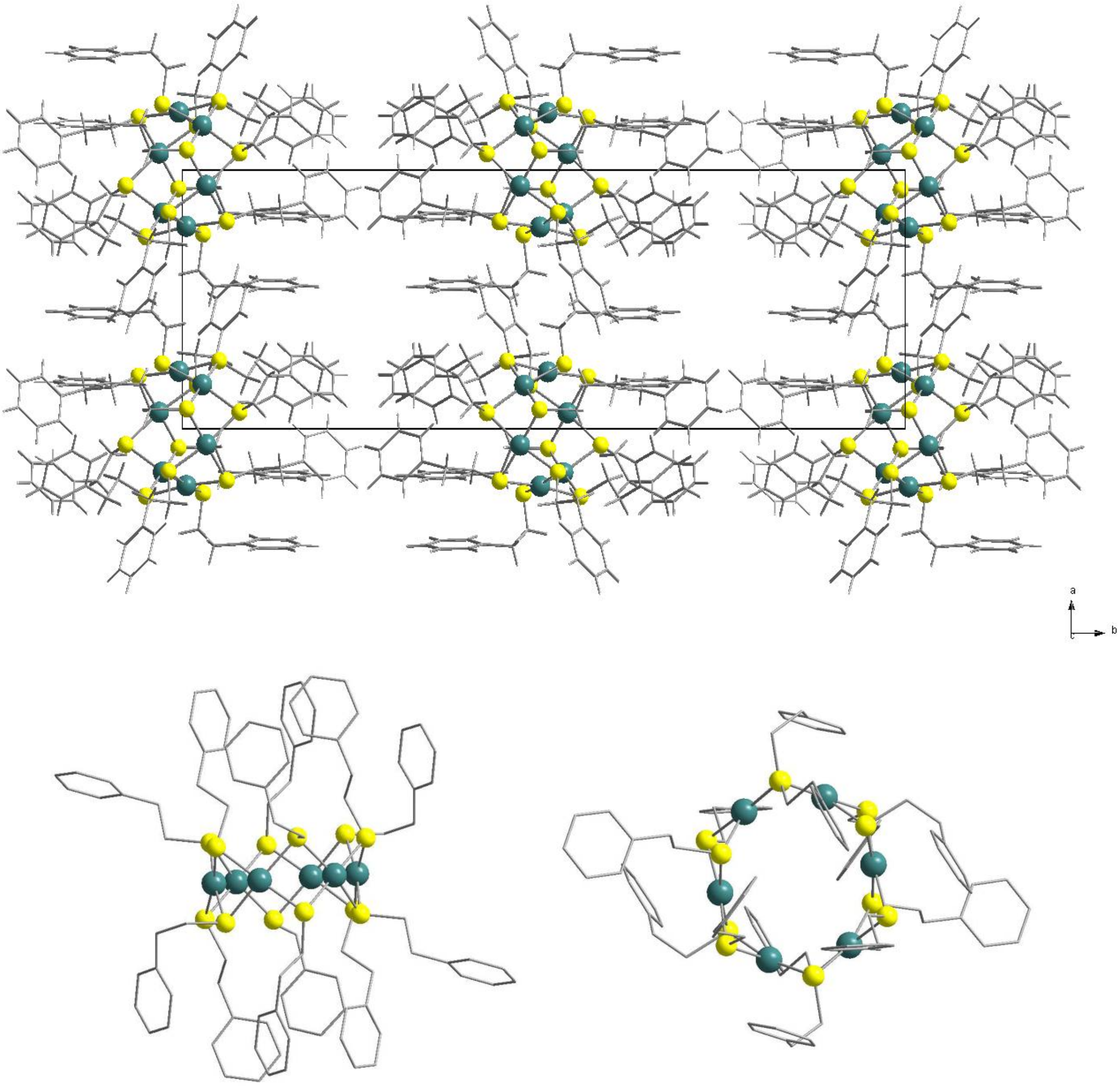

Figure S2 | The single crystal structure of $[Pd(SC_2H_4Ph)_2]_6$. Blue, Pd; yellow, S.

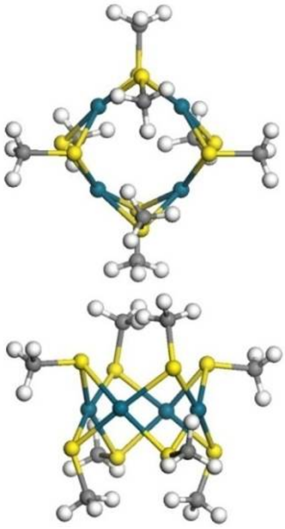

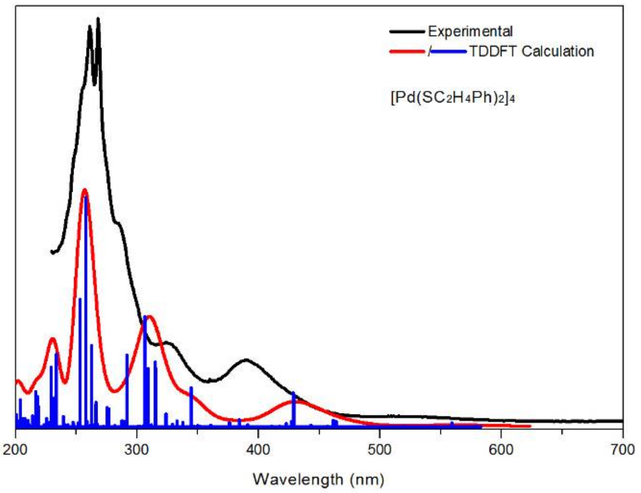
Experimental
/ TDDFT Calculation
[Pd(SC2H4Ph)2]4
200
300
400
500
600
700
Wavelength (nm)

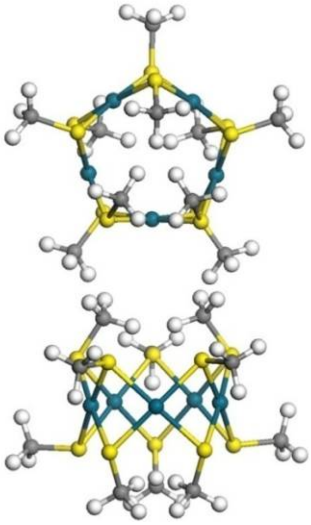

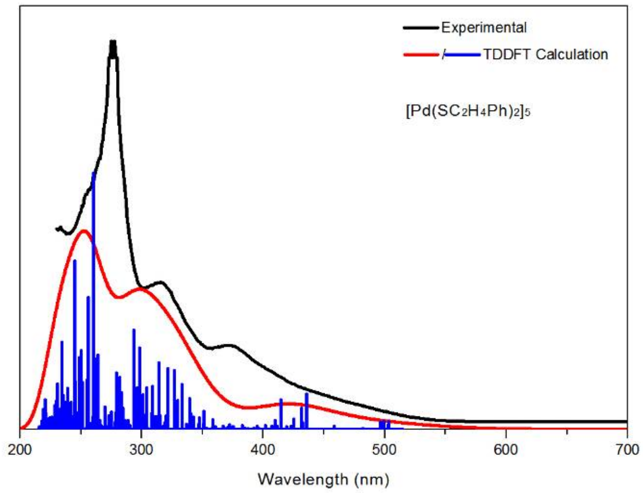
Experimental
/ TDDFT Calculation
[Pd(SC2H4Ph)2]5
200
300
400
500
600
700
Wavelength (nm)

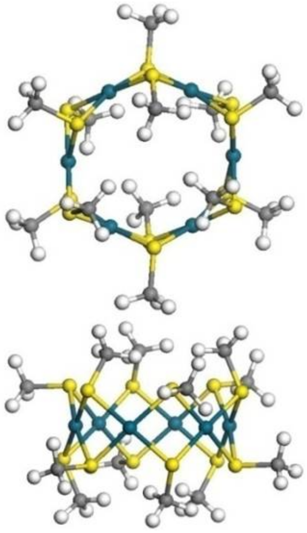

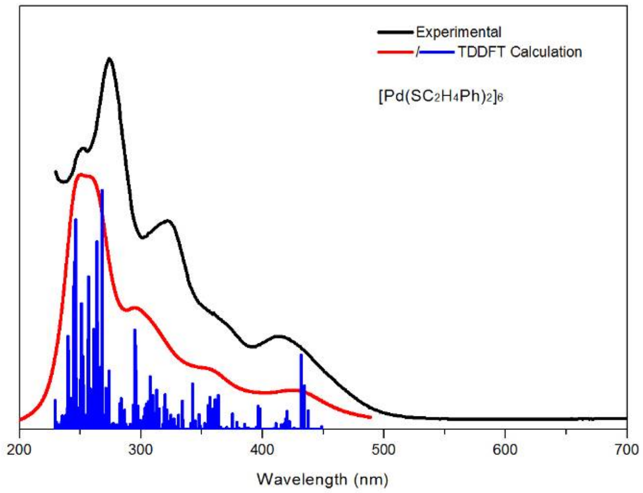
Experimental
/ TDDFT Calculation
[Pd(SC2H4Ph)2]6
200
300
400
500
600
700
Wavelength (nm)

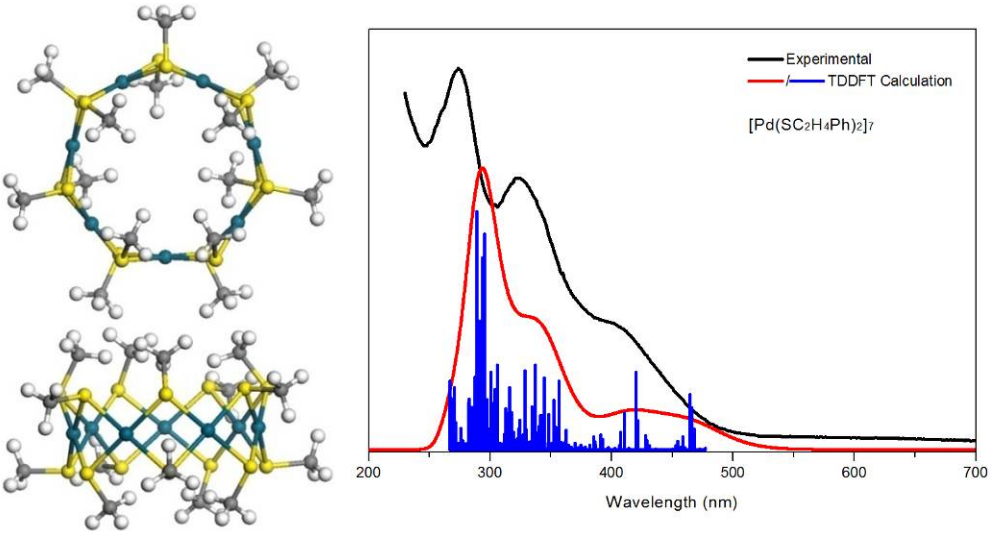
Experimental
TDDFT Calculation
$[Pd(SC_2H_4Ph)_2]_7$
200
300
400
500
600
700
Wavelength (nm)

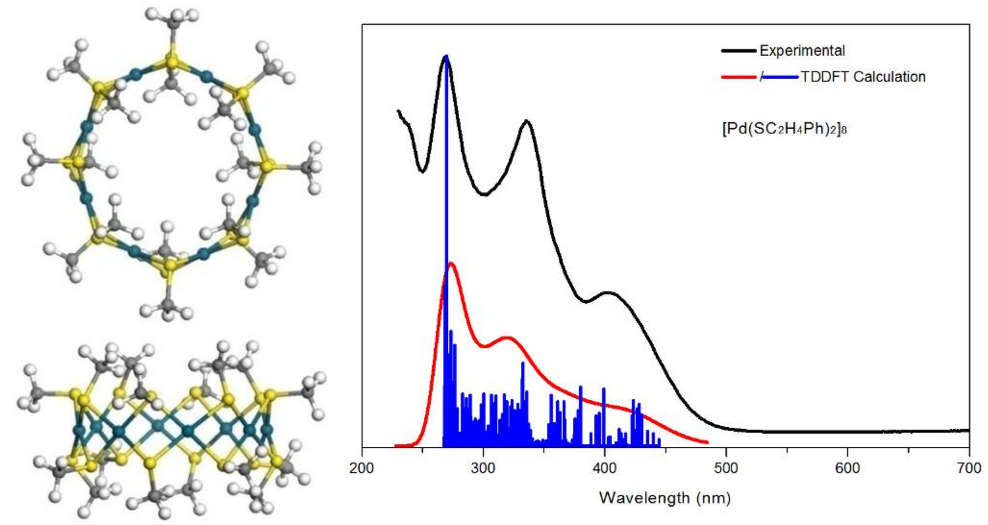
Experimental
TDDFT Calculation
$[Pd(SC_2H_4Ph)_2]_8$
200
300
400
500
600
700
Wavelength (nm)

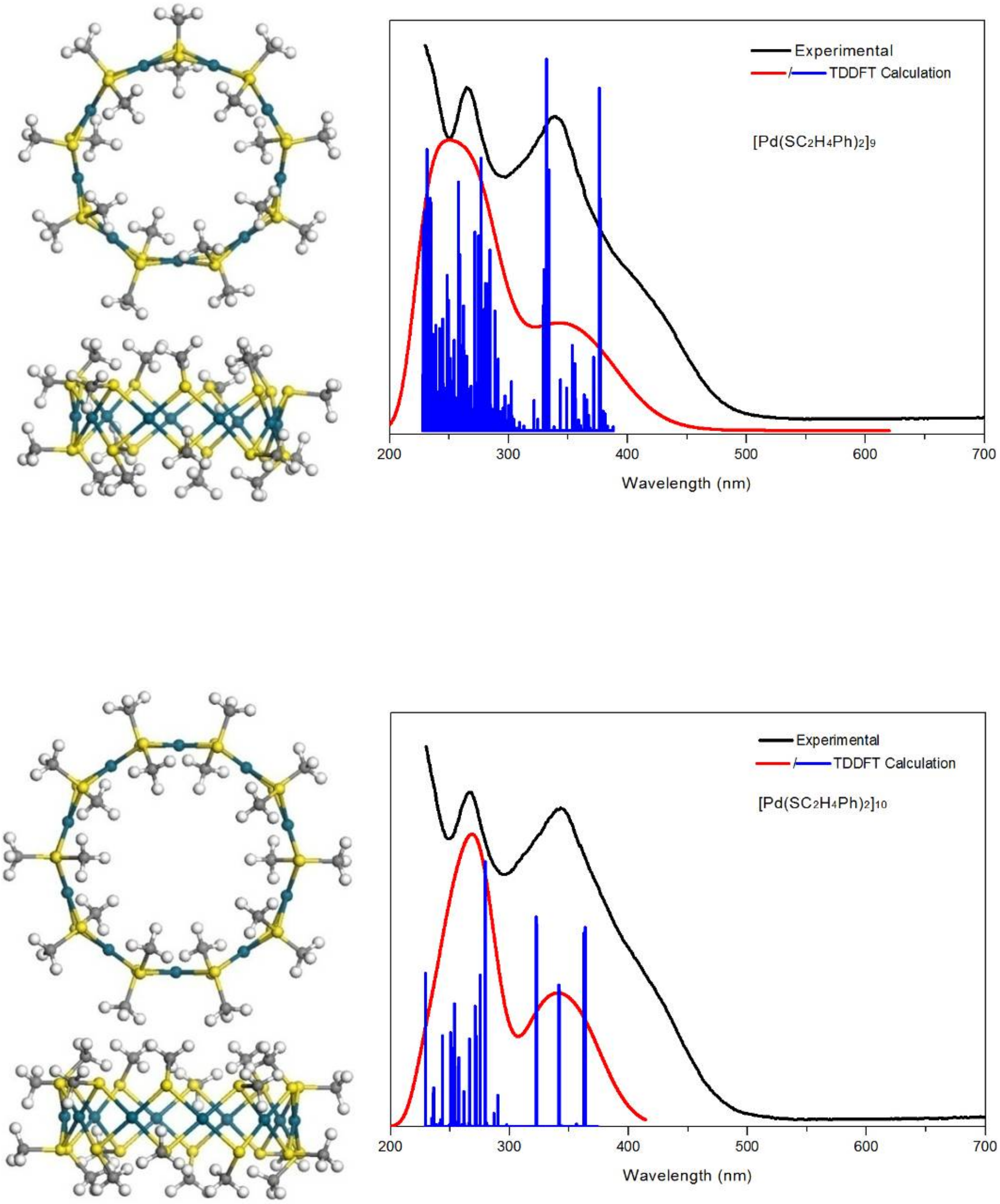

Figure S3 | Optimized molecular structure and optimized/experimental optical spectra of $[Pd(SCH_3)_2]_n$ ($4 \leq n \leq 10$). Blue, Pd; yellow, S; black, C; gray, H.

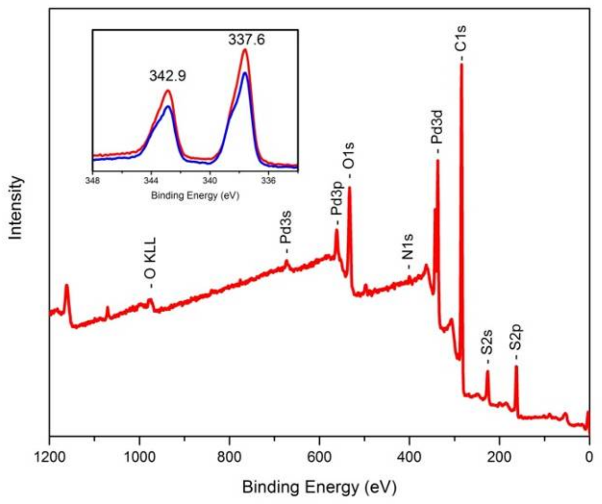

Figure S4 | XPS spectra of the product when $Pd(NO_3)_2$ reacts with 2-phenylethanethiol without (red) or with (blue) the addition of triethylamine.

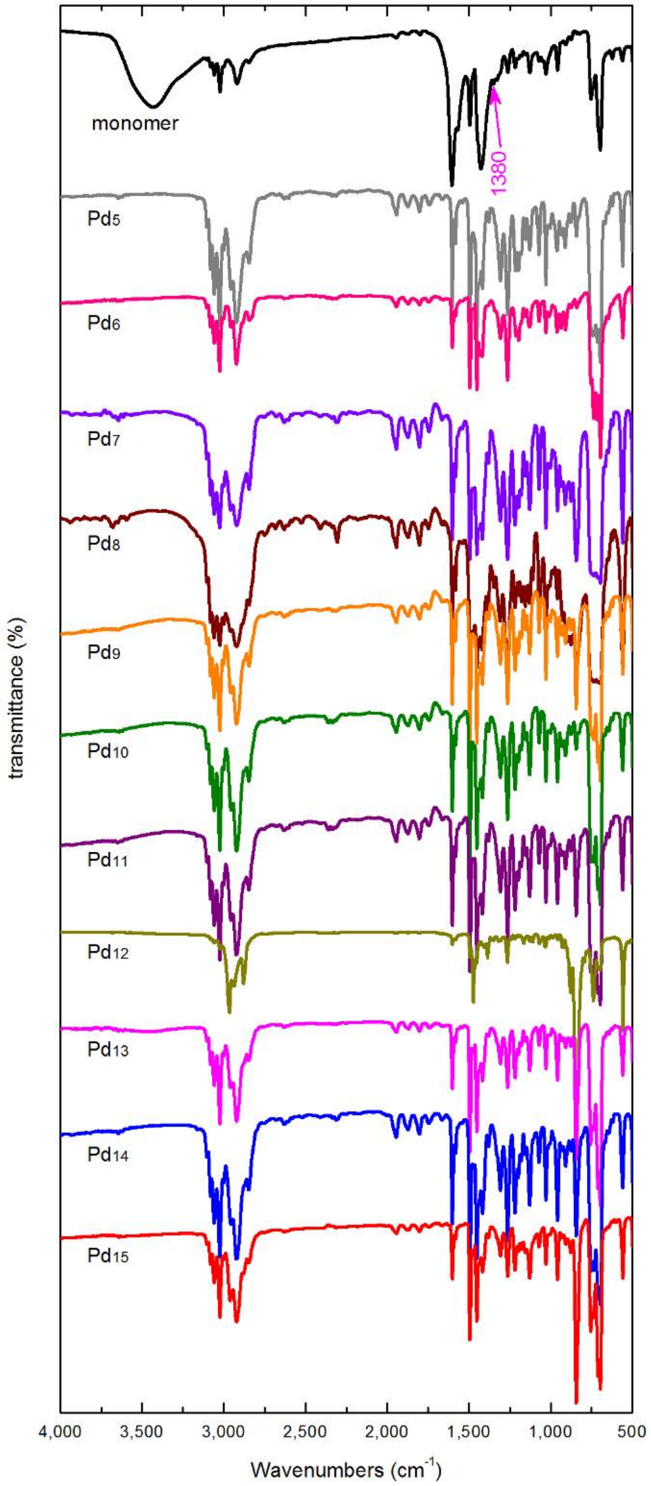

Figure S5 | FIIR absorption spectra of $PdH(SC_2H_4Ph)_2(NO_3)(H_2O)$ monomer and $[Pd(SC_2H_4Ph)_2]_n$ ($5 \leq n \leq 15$).

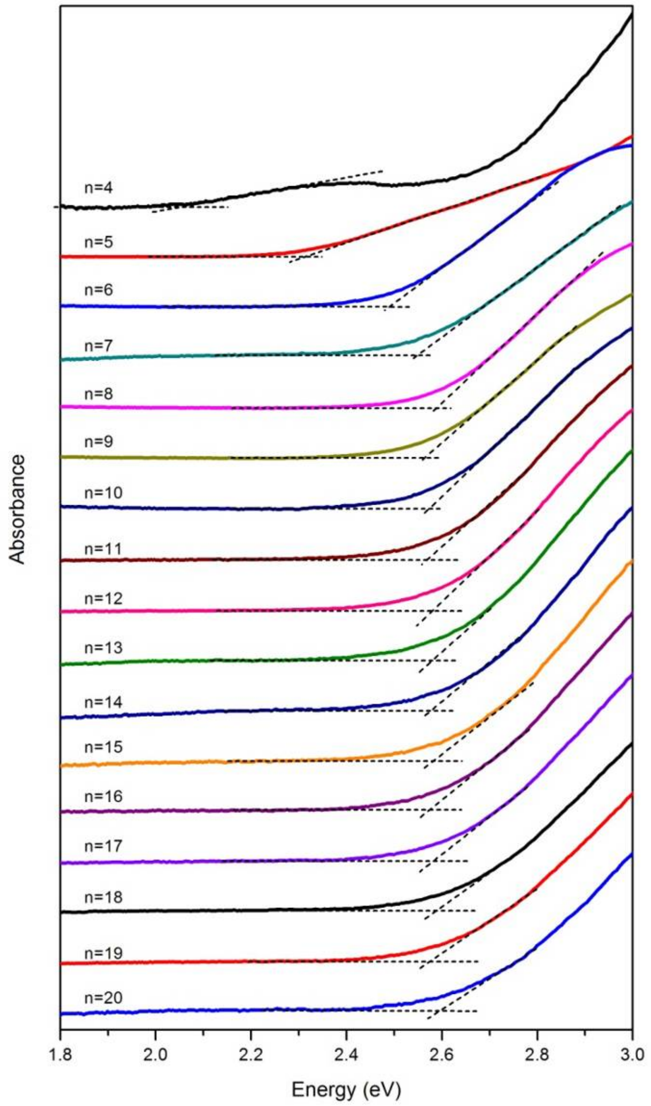

Figure S6 | Onsets in UV/Vis absorption spectra of $[Pd(SC_2H_4Ph)_2]_n$ ($4 \leq n \leq 20$).

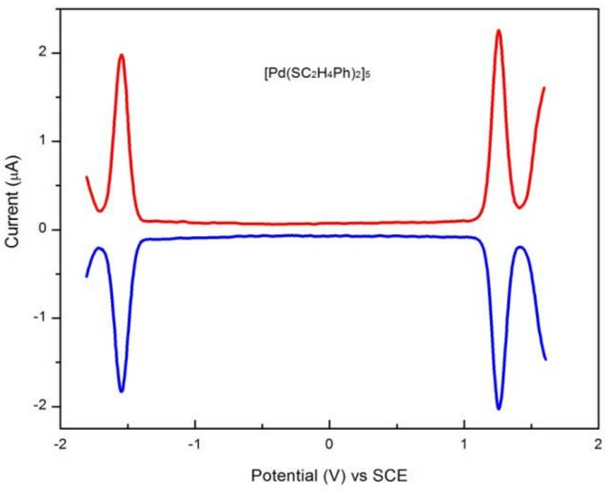
[Pd(SC2H4Ph)2]5
Current (μA)
Potential (V) vs SCE
-2
-1
0
1
2

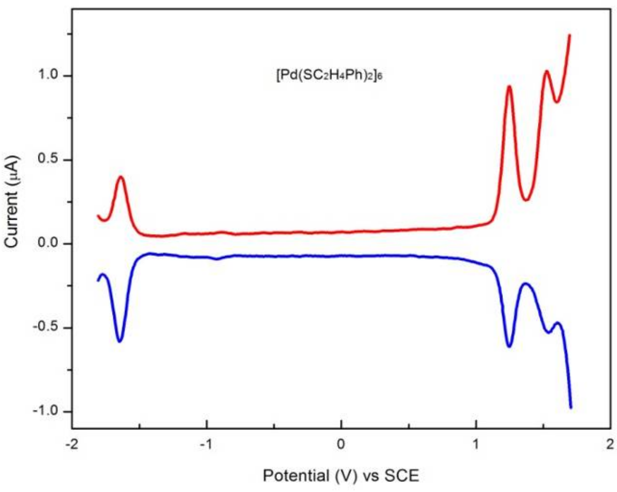
[Pd(SC2H4Ph)2]6
Current (μA)
Potential (V) vs SCE
1.0
0.5
0.0
-0.5
-1.0
-2
-1
0
1
2

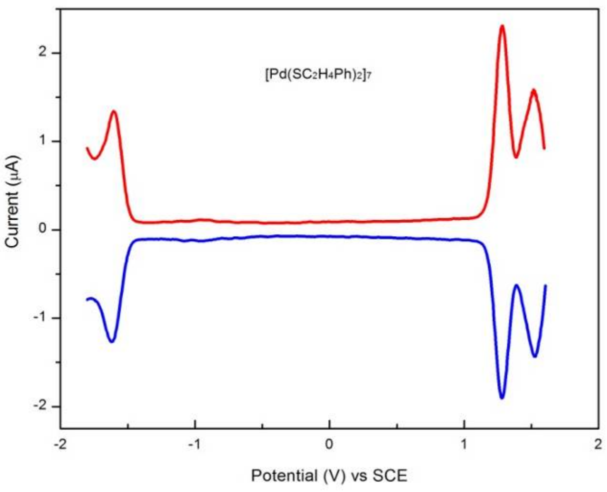
[Pd(SC2H4Ph)2]7
Current (μA)
Potential (V) vs SCE
-2
-1
0
1
2

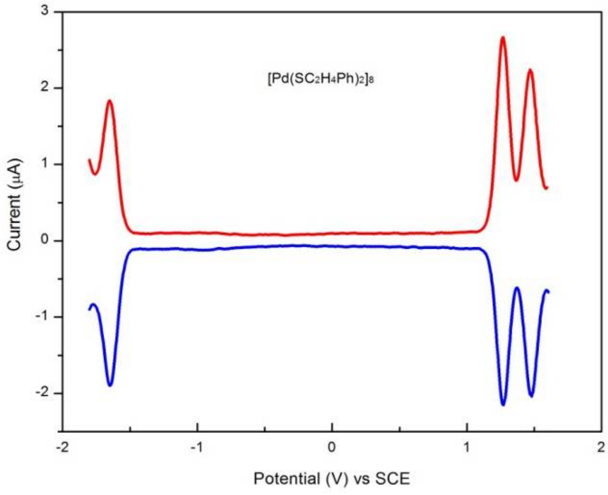
[Pd(SC2H4Ph)2]8
Current (μA)
Potential (V) vs SCE
3
2
1
0
-1
-2
-2
-1
0
1
2

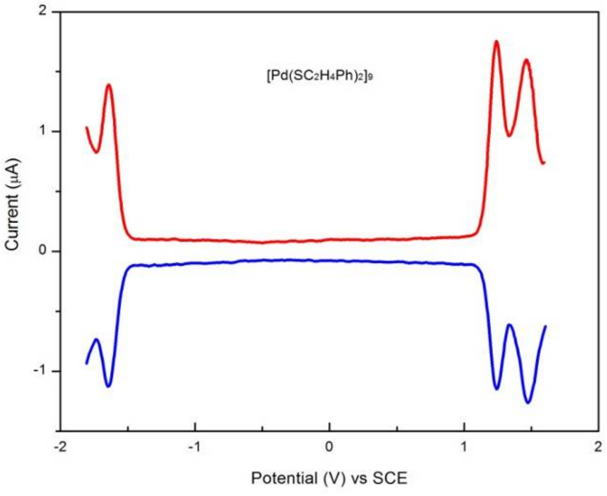
[Pd(SC2H4Ph)2]9
Current (μA)
Potential (V) vs SCE
2
1
0
-1
-2
-1
0
1
2

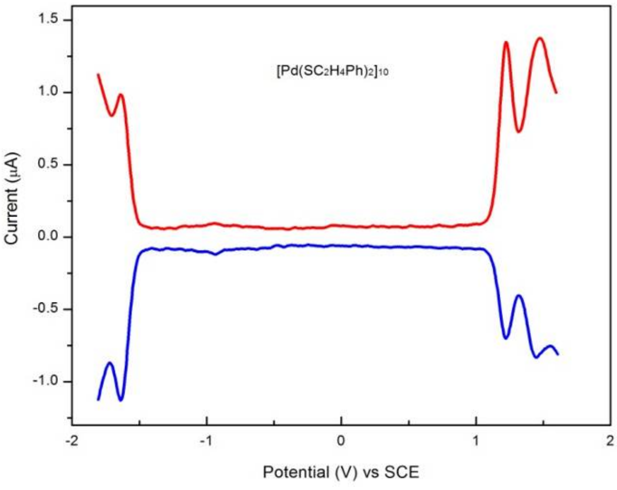
[Pd(SC2H4Ph)2]10
Current (μA)
Potential (V) vs SCE
1.5
1.0
0.5
0.0
-0.5
-1.0
-2
-1
0
1
2

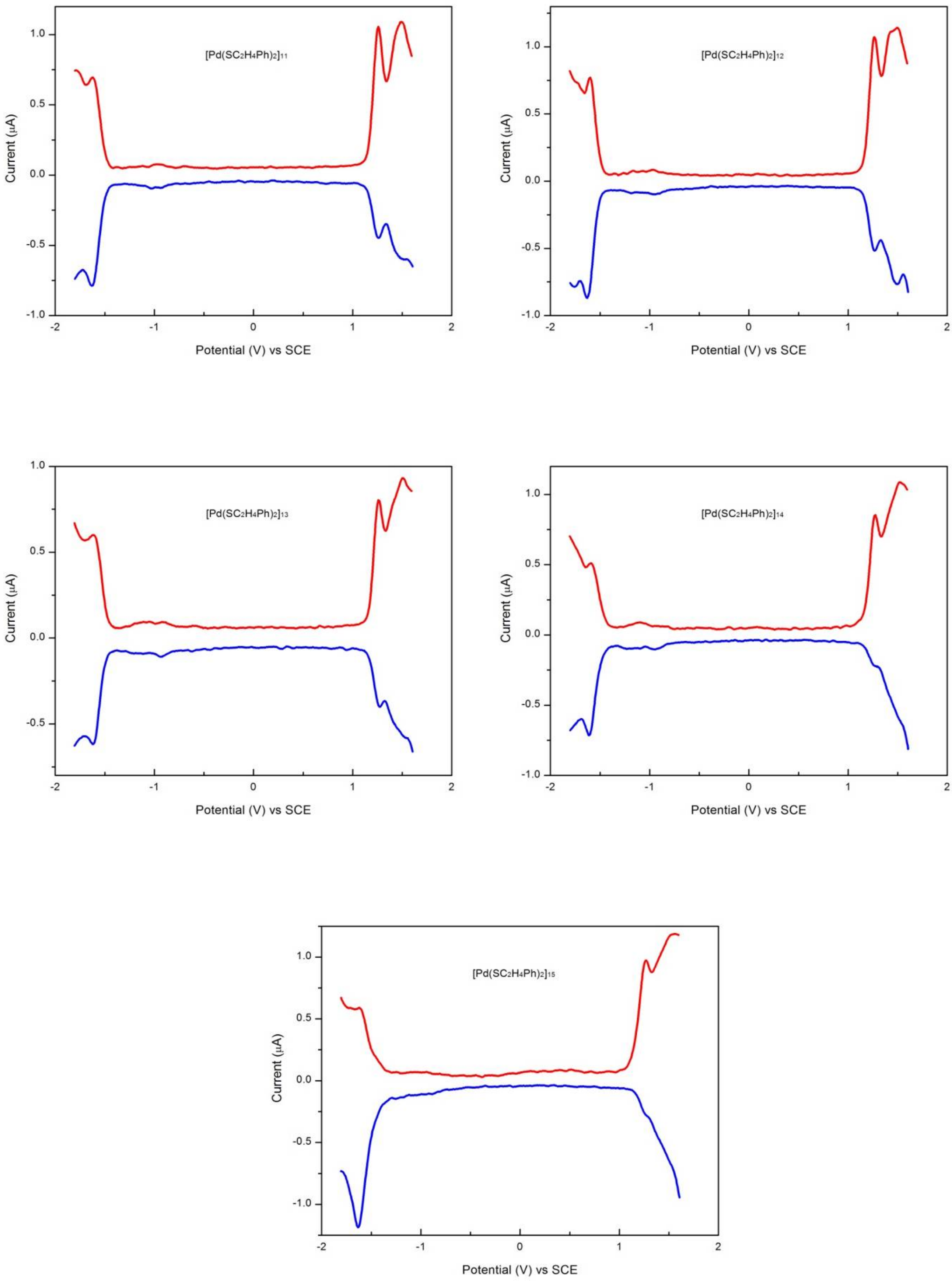

Figure S7 | Differential pulse voltammetry (DPV) of $[Pd(SC_2H_4Ph)_2]_n$, (5 ≤n ≤15).

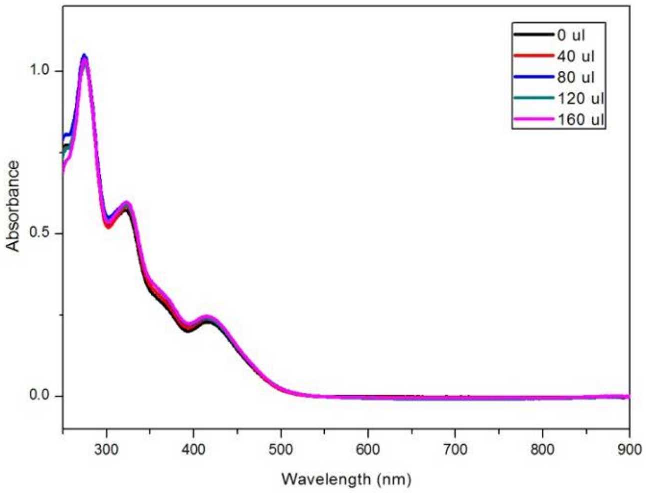

Figure S8 | UV/Vis absorption spectra of $[Pd(SC_2H_4Ph)_2]_6$ with the addition of $NaBH_4$ at room temperature under air atmosphere. Nanoclusters (0.1 mg, ca. 0.5×10^{-7} mol) was dissolved in 0.8 mL THF, and 2.5×10^{-2} M $NaBH_4$ aqueous solution (40 μL, 10×10^{-7} mol) was added at once for every 15 minutes.

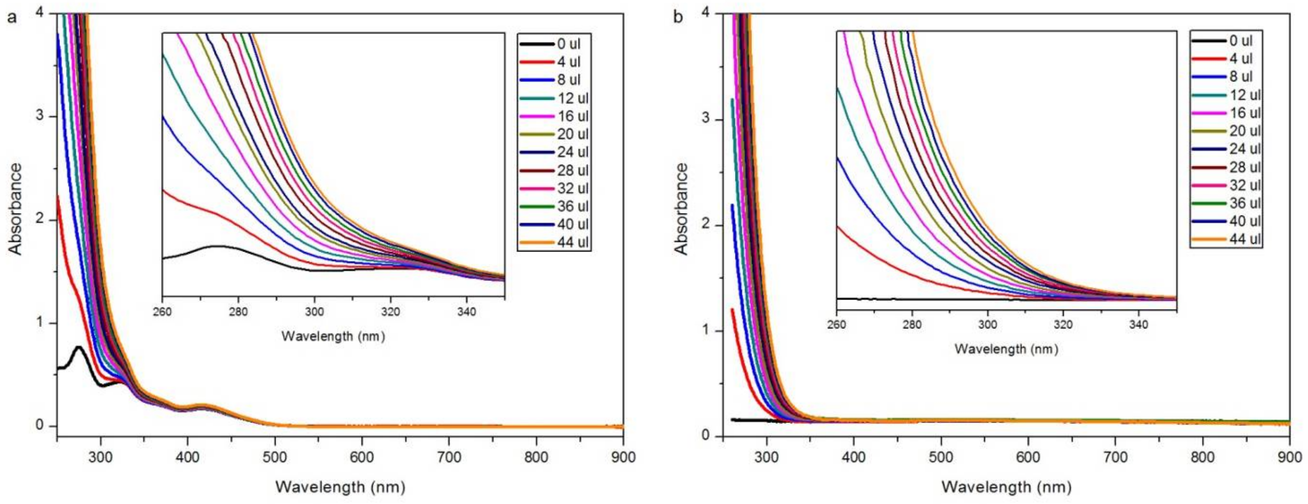

Figure S9 | UV/Vis absorption spectra of $[Pd(SC_2H_4Ph)_2]_6$ (a) and pure THF (b) with the addition of H_2O_2 at room temperature under air atmosphere. Nanoclusters (0.1 mg, about 5×10^{-5} mmol) was dissolved in 0.8 mL THF solution and 4 μL H_2O_2 (5.1×10^{-2} mmol) aqueous solution was added at once for every 6 minutes.

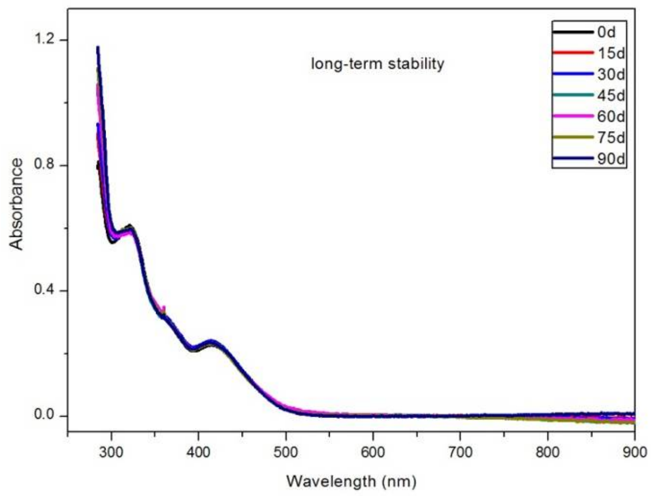

Figure S10 | UV/Vis absorption spectra of toluene solutions of $[Pd(SC_2H_4Ph)_2]_6$ at room temperature for different times under air atmosphere.

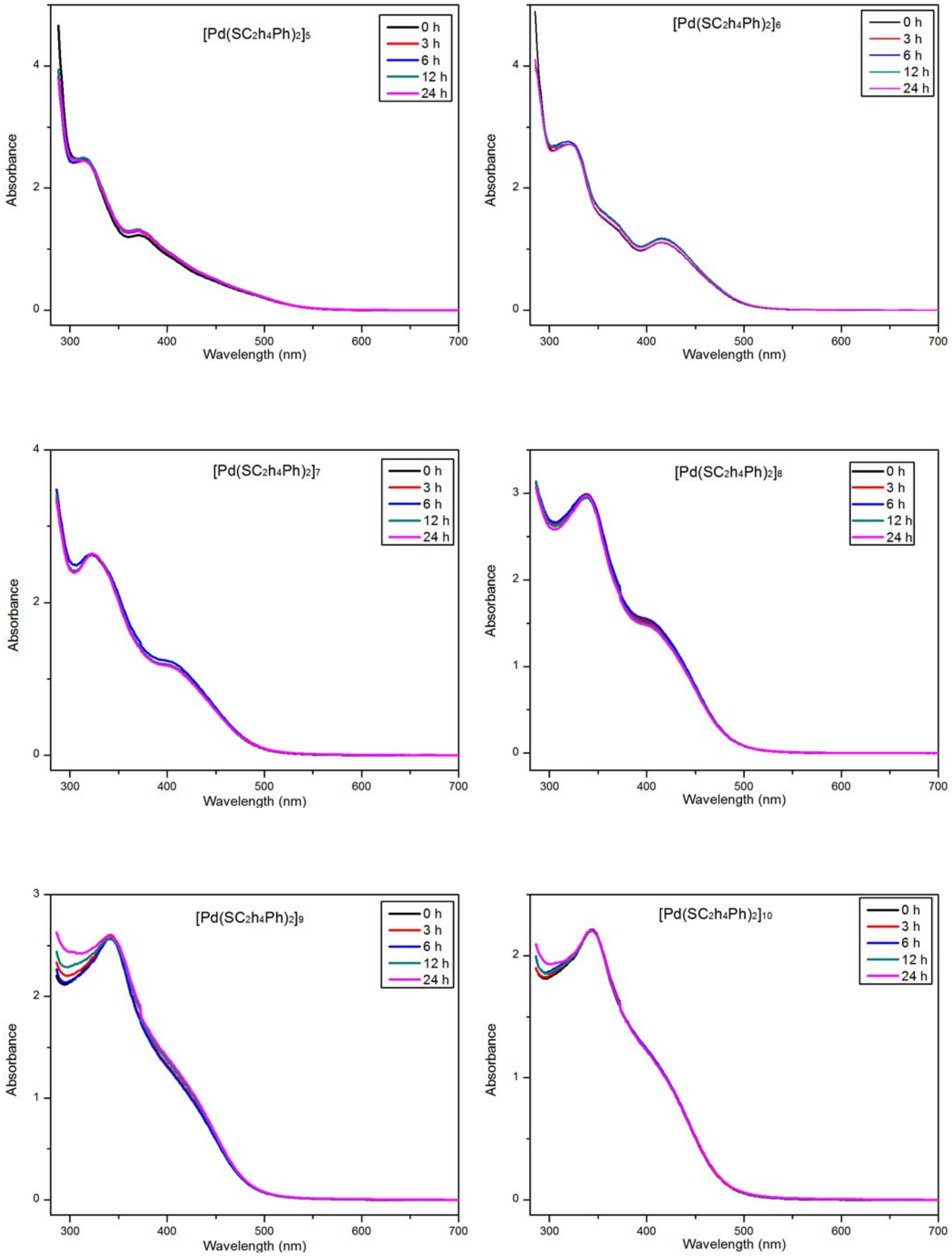
[Pd(SC2h4Ph)2]5
[Pd(SC2h4Ph)2]6
[Pd(SC2h4Ph)2]7
[Pd(SC2h4Ph)2]8
[Pd(SC2h4Ph)2]9
[Pd(SC2h4Ph)2]10
0 h
3 h
6 h
12 h
24 h
Absorbance
Wavelength (nm)

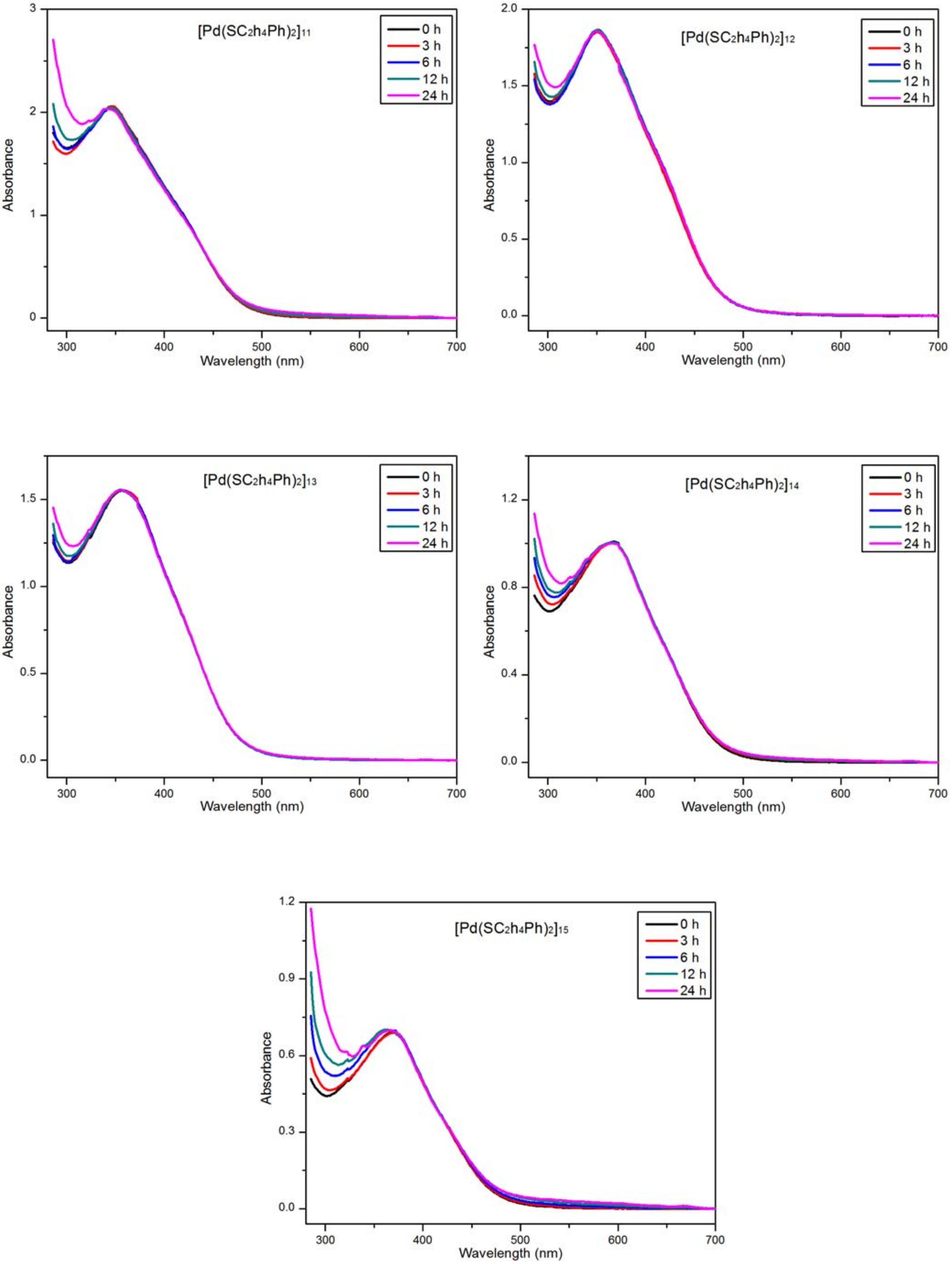

Figure S11 | UV/Vis absorption spectra of toluene solutions of $[Pd(SC_2H_4Ph)_2]_n$ at 80 ^oC for different times under air atmosphere.

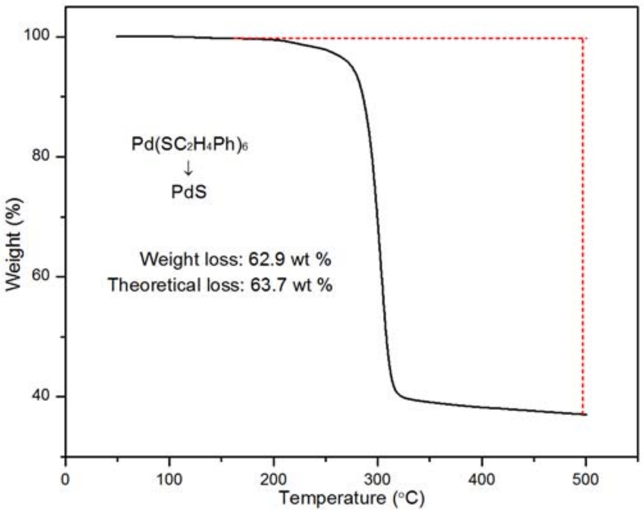

Figure S12 | TGA of $[Pd(SC_2H_4Ph)_2]_6$.

Table S1 | Summary of tiara-like $[M(SR)_2]_n$ (M = Ni, Pd, Pt) nanoclusters.

$[Ni(SR)_2]_n$	
n	SR
4	S*i*-Pr[S11], SC_6H_{11}[S12], SC_5H_9NMe[S13]
5	SEt[S12], SC_2H_4N(i-Pr)2[S14], SCH_2SiMe_3[S15]
6	SMe[S16, S17], SEt[S18, S19], S*n*-Pr[S20, S21], SEtOH[S22, S23], SCH_2CH_2S[S24], (SMe)/(S*i*-Pr) [S25], (SMe)/(S*t*-Bu)[S25], (SEt)/(S*i*-Pr)[S25], (S*i*-Pr)/(mtet) [S26], $S(CH_2)_3NMe_2$[S27], S(CH2)$_3$NHMe[S28], $S(CH_2)_2SC_6H_4Cl$[S29], SCH_2PhCl[S30], $SC_2H_4SiMe_3$[S31], SC_2H_4Ph[S32, S33]
8	SCH_2COOEt[S34]
9	SPh[S35]
10	(SEt)/(S*t*-Bu) [S25], (S*t*-Bu)/(pyet) [S36], (S*t*-Bu)/(atet) [S36], (S*t*-Bu)/(mtet)[S26]
11	SPh[S35]
12	(S*t*-Bu)/(etet) [S36]
$[Pd(SR)_2]_n$	
n	SR
6	SEt[S37], S*n*-Pr[S38, S39], S*n*-Bu[S40], SPh[S40], SHex[S41], $SC_{12}H_{25}$[S42], SCH_2COOMe[S43]
8	S*n*-Pr[S39], SCH_2COOMe[S44]
$[Pt(SR)_2]_n$	
n	SR
8	SCH_2COOMe[S44]

Notes: $(SR_1)/(SR_2)$: in the cluster contains two type thiols with number rate as 1/1;

SCH_2CH_2S is for $[Ni(SCH_2CH_2S)]_6$;

Me=CH_3;

Et=CH_2CH_3;

EtOH=CH_2CH_2OH;

n-Pr=$CH_2Ch_2CH_3$;

i-Pr=$CH(CH_3)_2$;

t-Bu=$C(CH_3)_3$;

Hex=$(CH_2)_5CH_3$;

pyet=2-(2-mercaptoethyl)pyridine;

atet=2-aminoethanethiol;

mtet =methylthioethanethiolate;

etet=2-ethylthioethanethiolate.

Table S2 | Summary of differential pulse voltammetry (DPV) and UV/vis absorption results of $[Pd(SC_2H_4Ph)_2]_n$ ($5 \leq n \leq 15$).

n value	The first reduction potential (V)	The first oxidation potential (V)	Electrochemical gap (V)	UV onset (eV)
				2.06
5	-1.536	1.257	2.793	2.32
6	-1.643	1.248	2.891	2.49
7	-1.62	1.275	2.895	2.55
8	-1.656	1.275	2.931	2.59
9	-1.635	1.244	2.879	2.56
10	-1.643	1.225	2.868	2.57
11	-1.627	1.262	2.889	2.57
12	-1.61	1.272	2.882	2.58
13	-1.625	1.255	2.88	2.58
14	-1.595	1.272	2.867	2.58
15	-1.621	1.267	2.888	2.58

Table S3 | Crystal data and structure refinement for $[Pd(SC_2H_4Ph)_2]_6$.

**Empirical formula**	$C_{108}H_{117}Pd_6S_{12}$	
**Formula weight**	2438.13	
**Temperature**	296(2) K	
**Wavelength**	0.71073 Å	
**Crystal system**	Monoclinic	
**Space group**	P 21/c	
**Unit cell dimensions**	a = 12.8908(7) Å	α= 90°.
	b = 35.953(2) Å	β= 90.3070(10)°.
	c = 11.2655(6) Å	γ= 90°.
**Volume**	5221.0(5) $Å^3$	
**Z**	2	
**Density (calculated)**	1.551 Mg/m^3	
**Absorption coefficient**	1.297 mm^{-1}	
**F(000)**	2466	
**Crystal size**	0.220 × 0.120 × 0.120 mm^3	
**Theta range for data collection**	1.133 to 26.009°.	
**Index ranges**	-15<=h<=15, -28<=k<=44, -13<=l<=13	
**Reflections collected**	27160	
**Independent reflections**	10182 [R(int) = 0.0311]	
**Completeness to theta = 25.242°**	99.3 %	
**Absorption correction**	None	
**Refinement method**	Full-matrix least-squares on F^2	
**Data / restraints / parameters**	10182 / 0 / 556	
**Goodness-of-fit on F^2**	1.111	
**Final R indices [I > 2 sigma(I)]**	R1 = 0.0363, wR2 = 0.0998	
**R indices (all data)**	R1 = 0.0472, wR2 = 0.1183	
**Extinction coefficient**	0.00134(10)	
**Largest diff. peak and hole**	1.556 and -0.882 e.$Å^{-3}$	

Table S4 | Atomic coordinates (× 10^4) and equivalent isotropic displacement parameters ($Å^2 \times 10^3$) for $[Pd(SC_2H_4Ph)_2]_6$. U(eq) is defined as one third of the trace of the orthogonalized U^{ij} tensor.

	x	y	z	U(eq)
Pd(1)	7792(1)	51(1)	4423(1)	15(1)
Pd(2)	9365(1)	328(1)	2450(1)	14(1)
Pd(3)	11677(1)	272(1)	3063(1)	14(1)
S(1)	7505(1)	284(1)	6331(1)	16(1)
S(2)	7984(1)	611(1)	3413(1)	17(1)
S(3)	10616(1)	788(1)	2760(1)	17(1)
S(4)	7390(1)	-508(1)	5352(1)	16(1)
S(5)	8314(1)	-195(1)	2611(1)	16(1)
S(6)	10732(1)	58(1)	1433(1)	16(1)
C(1)	6110(3)	221(1)	6493(4)	22(1)
C(2)	5479(3)	433(1)	5569(4)	24(1)
C(3)	5555(3)	851(1)	5562(4)	23(1)
C(4)	5733(4)	1054(2)	6575(5)	30(1)
C(5)	5701(4)	1443(2)	6572(5)	39(1)
C(6)	5511(4)	1630(2)	5534(6)	44(2)
C(7)	5374(4)	1434(2)	4498(6)	45(2)
C(8)	5391(4)	1049(2)	4516(5)	32(1)
C(9)	8403(4)	985(1)	4411(4)	22(1)
C(10)	8486(4)	1352(1)	3733(4)	25(1)
C(11)	8736(4)	1677(1)	4543(4)	23(1)
C(12)	8008(4)	1948(1)	4767(5)	33(1)
C(13)	8223(5)	2248(2)	5514(6)	44(2)
C(14)	9186(6)	2272(2)	6035(5)	49(2)
C(15)	9928(5)	2004(2)	5840(6)	48(2)
C(16)	9694(4)	1708(2)	5094(5)	34(1)
C(17)	10965(3)	978(1)	1311(4)	22(1)
C(18)	12006(4)	1182(1)	1362(4)	26(1)
C(19)	12034(4)	1499(1)	2236(4)	26(1)
C(20)	12782(4)	1509(2)	3134(5)	31(1)
C(21)	12794(5)	1800(2)	3948(5)	40(1)
C(22)	12077(5)	2087(2)	3878(5)	42(1)
C(23)	11345(5)	2081(2)	2986(6)	45(2)
C(24)	11324(4)	1794(2)	2173(5)	37(1)
C(25)	8030(3)	-893(1)	4612(4)	19(1)
C(26)	7302(4)	-1053(1)	3660(4)	23(1)
C(27)	7783(4)	-1339(1)	2861(4)	21(1)

C(28)	7293(4)	-1675(2)	2679(5)	33(1)
C(29)	7712(5)	-1939(2)	1907(5)	43(2)
C(30)	8642(4)	-1867(2)	1325(5)	34(1)
C(31)	9119(4)	-1530(2)	1509(4)	30(1)
C(32)	8699(4)	-1271(1)	2266(4)	25(1)
C(33)	7138(3)	-177(1)	1684(4)	20(1)
C(34)	7229(3)	-463(1)	678(4)	21(1)
C(35)	6188(3)	-549(1)	108(4)	22(1)
C(36)	5888(4)	-390(2)	-959(4)	29(1)
C(37)	4947(4)	-470(2)	-1481(4)	31(1)
C(38)	4278(4)	-716(2)	-943(5)	35(1)
C(39)	4555(4)	-874(2)	146(5)	42(2)
C(40)	5501(4)	-791(2)	643(5)	39(1)
C(41)	10697(3)	-448(1)	1414(4)	19(1)
C(42)	11666(3)	-590(1)	752(4)	18(1)
C(43)	11722(3)	-1011(1)	762(4)	20(1)
C(44)	11634(4)	-1216(1)	-281(4)	26(1)
C(45)	11664(4)	-1601(2)	-258(5)	34(1)
C(46)	11783(4)	-1787(2)	804(6)	39(1)
C(47)	11897(4)	-1589(2)	1836(5)	38(1)
C(48)	11860(4)	-1203(1)	1824(4)	27(1)
C(49)	5968(7)	2963(3)	6610(8)	73(2)
C(50)	4792(14)	2959(5)	6190(16)	74(5)
C(51)	4416(8)	2642(3)	6888(9)	87(3)
C(52)	4980(16)	2466(6)	7821(19)	95(6)
C(53)	5981(10)	2467(4)	8039(11)	47(3)
C(54)	6432(15)	2778(5)	7301(16)	76(5)
C(49')	5968(7)	2963(3)	6610(8)	73(2)
C(50')	5533(11)	2646(4)	7273(13)	55(4)
C(51')	4416(8)	2642(3)	6888(9)	87(3)
C(52')	4000(15)	2893(5)	5945(16)	74(5)
C(53')	4414(10)	3162(4)	5497(12)	50(4)
C(54')	5555(13)	3184(5)	5942(15)	73(5)

Table S5 | Bond lengths [Å] and angles [°] for $[Pd(SC_2H_4Ph)_2]_6$.

Pd(1)-S(2)	2.3252(11)	C(14)-C(15)	1.376(9)
Pd(1)-S(4)	2.3252(11)	C(15)-C(16)	1.388(8)
Pd(1)-S(5)	2.3278(11)	C(17)-C(18)	1.530(6)
Pd(1)-S(1)	2.3385(11)	C(18)-C(19)	1.504(7)
Pd(1)-Pd(3)#1	3.1342(5)	C(19)-C(20)	1.394(7)
Pd(1)-Pd(2)	3.1765(5)	C(19)-C(24)	1.402(7)
Pd(2)-S(6)	2.3201(11)	C(20)-C(21)	1.390(8)
Pd(2)-S(2)	2.3235(11)	C(21)-C(22)	1.389(8)
Pd(2)-S(5)	2.3277(11)	C(22)-C(23)	1.376(8)
Pd(2)-S(3)	2.3335(11)	C(23)-C(24)	1.381(8)
Pd(2)-Pd(3)	3.0618(5)	C(25)-C(26)	1.533(6)
Pd(3)-S(4)#1	2.3090(10)	C(26)-C(27)	1.503(6)
Pd(3)-S(6)	2.3281(11)	C(27)-C(28)	1.378(7)
Pd(3)-S(3)	2.3288(11)	C(27)-C(32)	1.383(7)
Pd(3)-S(1)#1	2.3587(11)	C(28)-C(29)	1.398(7)
Pd(3)-Pd(1)#1	3.1342(5)	C(29)-C(30)	1.394(8)
S(1)-C(1)	1.823(4)	C(30)-C(31)	1.374(7)
S(1)-Pd(3)#1	2.3588(11)	C(31)-C(32)	1.375(7)
S(2)-C(9)	1.833(4)	C(33)-C(34)	1.534(6)
S(3)-C(17)	1.829(5)	C(34)-C(35)	1.516(6)
S(4)-C(25)	1.817(5)	C(35)-C(40)	1.383(7)
S(4)-Pd(3)#1	2.3090(10)	C(35)-C(36)	1.385(7)
S(5)-C(33)	1.837(4)	C(36)-C(37)	1.377(7)
S(6)-C(41)	1.821(5)	C(37)-C(38)	1.378(8)
C(1)-C(2)	1.522(6)	C(38)-C(39)	1.396(7)
C(2)-C(3)	1.507(7)	C(39)-C(40)	1.372(7)
C(3)-C(4)	1.372(7)	C(41)-C(42)	1.544(6)
C(3)-C(8)	1.392(7)	C(42)-C(43)	1.518(6)
C(4)-C(5)	1.398(8)	C(43)-C(44)	1.390(6)
C(5)-C(6)	1.371(9)	C(43)-C(48)	1.392(7)
C(6)-C(7)	1.374(9)	C(44)-C(45)	1.384(7)
C(7)-C(8)	1.385(8)	C(45)-C(46)	1.379(8)
C(9)-C(10)	1.528(7)	C(46)-C(47)	1.370(8)
C(10)-C(11)	1.516(7)	C(47)-C(48)	1.390(7)
C(11)-C(12)	1.376(7)	C(49)-C(54)	1.183(18)
C(11)-C(16)	1.384(7)	C(49)-C(50)	1.59(2)
C(12)-C(13)	1.396(8)	C(50)-C(51)	1.47(2)
C(13)-C(14)	1.374(9)	C(51)-C(52)	1.42(2)

C(52)-C(53)	1.31(2)
C(53)-C(54)	1.51(2)
C(52')-C(53')	1.22(2)
C(53')-C(54')	1.55(2)
S(2)-Pd(1)-S(4)	172.99(4)
S(2)-Pd(1)-S(5)	82.46(4)
S(4)-Pd(1)-S(5)	97.57(4)
S(2)-Pd(1)-S(1)	99.09(4)
S(4)-Pd(1)-S(1)	81.86(4)
S(5)-Pd(1)-S(1)	172.00(4)
S(2)-Pd(1)-Pd(3)#1	137.69(3)
S(4)-Pd(1)-Pd(3)#1	47.22(3)
S(5)-Pd(1)-Pd(3)#1	126.04(3)
S(1)-Pd(1)-Pd(3)#1	48.42(3)
S(2)-Pd(1)-Pd(2)	46.88(3)
S(4)-Pd(1)-Pd(2)	136.92(3)
S(5)-Pd(1)-Pd(2)	46.97(3)
S(1)-Pd(1)-Pd(2)	129.47(3)
Pd(3)#1-Pd(1)-Pd(2)	127.672(13)
S(6)-Pd(2)-S(2)	178.07(4)
S(6)-Pd(2)-S(5)	98.28(4)
S(2)-Pd(2)-S(5)	82.50(4)
S(6)-Pd(2)-S(3)	81.03(4)
S(2)-Pd(2)-S(3)	98.69(4)
S(5)-Pd(2)-S(3)	164.43(4)
S(6)-Pd(2)-Pd(3)	48.91(3)
S(2)-Pd(2)-Pd(3)	132.14(3)
S(5)-Pd(2)-Pd(3)	119.68(3)
S(3)-Pd(2)-Pd(3)	48.88(3)
S(6)-Pd(2)-Pd(1)	134.73(3)
S(2)-Pd(2)-Pd(1)	46.92(3)
S(5)-Pd(2)-Pd(1)	46.98(3)
S(3)-Pd(2)-Pd(1)	124.10(3)
Pd(3)-Pd(2)-Pd(1)	116.472(13)
S(4)#1-Pd(3)-S(6)	177.69(4)
S(4)#1-Pd(3)-S(3)	97.12(4)
S(6)-Pd(3)-S(3)	80.97(4)
S(4)#1-Pd(3)-S(1)#1	81.77(4)
S(6)-Pd(3)-S(1)#1	100.38(4)
S(3)-Pd(3)-S(1)#1	168.13(4)
S(4)#1-Pd(3)-Pd(2)	130.72(3)
S(6)-Pd(3)-Pd(2)	48.69(3)
S(3)-Pd(3)-Pd(2)	49.02(3)
S(1)#1-Pd(3)-Pd(2)	123.69(3)
S(4)#1-Pd(3)-Pd(1)#1	47.67(3)
S(6)-Pd(3)-Pd(1)#1	134.55(3)
S(3)-Pd(3)-Pd(1)#1	123.58(3)
S(1)#1-Pd(3)-Pd(1)#1	47.87(3)
Pd(2)-Pd(3)-Pd(1)#1	115.855(13)
C(1)-S(1)-Pd(1)	102.06(16)
C(1)-S(1)-Pd(3)#1	107.82(16)
Pd(1)-S(1)-Pd(3)#1	83.71(4)
C(9)-S(2)-Pd(2)	112.51(15)
C(9)-S(2)-Pd(1)	111.56(15)
Pd(2)-S(2)-Pd(1)	86.20(4)
C(17)-S(3)-Pd(3)	106.36(16)
C(17)-S(3)-Pd(2)	107.74(15)
Pd(3)-S(3)-Pd(2)	82.10(4)
C(25)-S(4)-Pd(3)#1	113.51(14)
C(25)-S(4)-Pd(1)	110.49(15)
Pd(3)#1-S(4)-Pd(1)	85.11(4)
C(33)-S(5)-Pd(2)	113.95(16)
C(33)-S(5)-Pd(1)	104.06(15)
Pd(2)-S(5)-Pd(1)	86.05(4)
C(41)-S(6)-Pd(2)	113.90(15)
C(41)-S(6)-Pd(3)	110.61(15)
Pd(2)-S(6)-Pd(3)	82.40(4)
C(2)-C(1)-S(1)	113.1(3)
C(3)-C(2)-C(1)	118.0(4)
C(4)-C(3)-C(8)	117.2(5)
C(4)-C(3)-C(2)	122.4(4)
C(8)-C(3)-C(2)	120.3(5)
C(3)-C(4)-C(5)	121.6(5)
C(6)-C(5)-C(4)	120.0(5)
C(5)-C(6)-C(7)	119.5(6)
C(6)-C(7)-C(8)	119.9(6)
C(7)-C(8)-C(3)	121.7(5)
C(10)-C(9)-S(2)	110.4(3)
C(11)-C(10)-C(9)	112.3(4)

C(12)-C(11)-C(16)	117.9(5)
C(12)-C(11)-C(10)	120.9(4)
C(16)-C(11)-C(10)	121.2(4)
C(11)-C(12)-C(13)	121.7(5)
C(14)-C(13)-C(12)	118.8(6)
C(13)-C(14)-C(15)	121.0(6)
C(14)-C(15)-C(16)	119.0(6)
C(11)-C(16)-C(15)	121.6(5)
C(18)-C(17)-S(3)	111.5(3)
C(19)-C(18)-C(17)	113.9(4)
C(20)-C(19)-C(24)	117.7(5)
C(20)-C(19)-C(18)	120.7(5)
C(24)-C(19)-C(18)	121.6(4)
C(21)-C(20)-C(19)	120.3(5)
C(22)-C(21)-C(20)	121.1(5)
C(23)-C(22)-C(21)	118.9(5)
C(22)-C(23)-C(24)	120.5(6)
C(23)-C(24)-C(19)	121.5(5)
C(26)-C(25)-S(4)	109.2(3)
C(27)-C(26)-C(25)	115.0(4)
C(28)-C(27)-C(32)	118.4(5)
C(28)-C(27)-C(26)	119.9(4)
C(32)-C(27)-C(26)	121.7(4)
C(27)-C(28)-C(29)	120.6(5)
C(30)-C(29)-C(28)	120.2(5)
C(31)-C(30)-C(29)	118.5(5)
C(30)-C(31)-C(32)	120.9(5)
C(31)-C(32)-C(27)	121.3(5)

C(34)-C(33)-S(5)	109.3(3)
C(35)-C(34)-C(33)	112.2(4)
C(40)-C(35)-C(36)	117.6(4)
C(40)-C(35)-C(34)	120.7(4)
C(36)-C(35)-C(34)	121.7(4)
C(37)-C(36)-C(35)	121.7(5)
C(36)-C(37)-C(38)	119.9(5)
C(37)-C(38)-C(39)	119.4(5)
C(40)-C(39)-C(38)	119.5(5)
C(39)-C(40)-C(35)	121.9(5)
C(42)-C(41)-S(6)	108.4(3)
C(43)-C(42)-C(41)	111.4(4)
C(44)-C(43)-C(48)	118.2(4)
C(44)-C(43)-C(42)	121.3(4)
C(48)-C(43)-C(42)	120.5(4)
C(45)-C(44)-C(43)	120.8(5)
C(46)-C(45)-C(44)	120.3(5)
C(47)-C(46)-C(45)	119.7(5)
C(46)-C(47)-C(48)	120.5(5)
C(47)-C(48)-C(43)	120.5(5)
C(54)-C(49)-C(50)	132.0(14)
C(51)-C(50)-C(49)	99.5(12)
C(52)-C(51)-C(50)	124.9(14)
C(53)-C(52)-C(51)	129(2)
C(52)-C(53)-C(54)	106.3(15)
C(49)-C(54)-C(53)	125.7(16)
C(52')-C(53')-C(54')	108.9(15)

Symmetry transformations used to generate equivalent atoms:

#1 -x+2,-y,-z+1

Table S6 | Anisotropic displacement parameters (Å^2 × 10^3) for $[Pd(SC_2H_4Ph)_2]_6$.
The anisotropic displacement factor exponent takes the form: $-2\pi^2[h^2 a*^2U^{11} + ... + 2 h k a* b* U^{12}]$

	U^{11}	U^{22}	U^{33}	U^{23}	U^{13}	U^{12}
Pd(1)	15(1)	14(1)	15(1)	0(1)	-1(1)	0(1)
Pd(2)	13(1)	15(1)	15(1)	0(1)	-1(1)	0(1)
Pd(3)	14(1)	15(1)	15(1)	0(1)	-2(1)	0(1)
S(1)	16(1)	16(1)	17(1)	-2(1)	-2(1)	2(1)
S(2)	18(1)	15(1)	18(1)	0(1)	1(1)	2(1)
S(3)	16(1)	16(1)	19(1)	0(1)	-1(1)	0(1)
S(4)	16(1)	16(1)	17(1)	-2(1)	-3(1)	0(1)
S(5)	16(1)	16(1)	17(1)	-1(1)	0(1)	-1(1)
S(6)	15(1)	17(1)	16(1)	-2(1)	-1(1)	1(1)
C(1)	16(2)	24(2)	27(2)	3(2)	2(2)	2(2)
C(2)	16(2)	27(3)	28(2)	-2(2)	-2(2)	5(2)
C(3)	13(2)	30(3)	26(2)	5(2)	1(2)	1(2)
C(4)	28(3)	32(3)	29(3)	-1(2)	-5(2)	2(2)
C(5)	38(3)	31(3)	48(3)	-11(3)	-5(3)	4(2)
C(6)	30(3)	29(3)	73(4)	5(3)	1(3)	2(2)
C(7)	37(3)	47(4)	50(4)	24(3)	0(3)	-1(3)
C(8)	27(3)	37(3)	33(3)	6(2)	1(2)	-1(2)
C(9)	28(2)	17(2)	20(2)	-5(2)	1(2)	3(2)
C(10)	29(2)	20(2)	27(2)	0(2)	0(2)	3(2)
C(11)	30(2)	14(2)	26(2)	2(2)	1(2)	-1(2)
C(12)	34(3)	22(3)	41(3)	1(2)	2(2)	2(2)
C(13)	63(4)	20(3)	49(4)	-1(3)	9(3)	5(3)
C(14)	81(5)	28(3)	37(3)	-6(3)	-6(3)	-12(3)
C(15)	52(4)	47(4)	44(4)	1(3)	-14(3)	-13(3)
C(16)	31(3)	28(3)	43(3)	2(2)	-6(2)	1(2)
C(17)	23(2)	21(2)	21(2)	5(2)	-6(2)	-3(2)
C(18)	24(2)	24(3)	29(3)	6(2)	1(2)	-4(2)
C(19)	26(2)	22(3)	29(3)	5(2)	3(2)	-6(2)
C(20)	29(3)	24(3)	39(3)	5(2)	-5(2)	-2(2)
C(21)	48(3)	36(3)	36(3)	3(3)	-11(3)	-11(3)
C(22)	60(4)	26(3)	41(3)	-4(3)	-4(3)	-6(3)
C(23)	48(3)	28(3)	58(4)	-7(3)	-9(3)	6(3)
C(24)	38(3)	30(3)	44(3)	-2(3)	-11(2)	7(2)
C(25)	20(2)	18(2)	19(2)	-2(2)	-4(2)	-2(2)
C(26)	22(2)	21(2)	26(2)	-4(2)	-4(2)	-4(2)

C(27)	24(2)	19(2)	20(2)	-2(2)	-5(2)	2(2)
C(28)	29(3)	27(3)	43(3)	-12(2)	9(2)	-7(2)
C(29)	52(4)	29(3)	49(4)	-19(3)	11(3)	-12(3)
C(30)	41(3)	28(3)	35(3)	-15(2)	4(2)	6(2)
C(31)	31(3)	33(3)	26(3)	0(2)	2(2)	3(2)
C(32)	27(2)	22(3)	27(2)	1(2)	-3(2)	-4(2)
C(33)	15(2)	26(2)	21(2)	0(2)	-2(2)	-3(2)
C(34)	19(2)	28(3)	16(2)	2(2)	2(2)	-6(2)
C(35)	18(2)	28(3)	19(2)	-4(2)	-3(2)	1(2)
C(36)	27(2)	34(3)	25(2)	9(2)	0(2)	-9(2)
C(37)	27(3)	43(3)	23(2)	8(2)	-6(2)	0(2)
C(38)	24(2)	51(4)	30(3)	0(3)	-10(2)	-6(2)
C(39)	32(3)	52(4)	41(3)	20(3)	-5(2)	-24(3)
C(40)	32(3)	56(4)	27(3)	13(3)	-10(2)	-10(3)
C(41)	16(2)	19(2)	23(2)	-4(2)	-3(2)	2(2)
C(42)	20(2)	17(2)	18(2)	0(2)	1(2)	2(2)
C(43)	16(2)	20(2)	23(2)	-4(2)	2(2)	2(2)
C(44)	22(2)	31(3)	24(2)	-9(2)	-1(2)	3(2)
C(45)	34(3)	26(3)	43(3)	-14(2)	2(2)	3(2)
C(46)	37(3)	22(3)	58(4)	-4(3)	-5(3)	5(2)
C(47)	41(3)	32(3)	42(3)	10(3)	-7(3)	8(3)
C(48)	31(3)	25(3)	24(2)	-6(2)	-5(2)	4(2)

Table S7 | Hydrogen coordinates (× 10^4) and isotropic displacement parameters ($Å^2$ × 10^3) for $[Pd(SC_2H_4Ph)_2]_6$.

	x	y	z	U(eq)
H(1A)	5948	-42	6431	27
H(1B)	5906	303	7278	27
H(2A)	5684	344	4792	29
H(2B)	4756	367	5673	29
H(4)	5878	929	7281	36
H(5)	5809	1574	7274	47
H(6)	5475	1889	5532	53
H(7)	5270	1560	3786	54
H(8)	5290	919	3810	39
H(9A)	7908	1012	5051	26
H(9B)	9072	923	4756	26
H(10A)	9024	1330	3138	30
H(10B)	7835	1400	3325	30
H(12)	7357	1930	4412	39
H(13)	7722	2429	5656	53
H(14)	9339	2473	6527	58
H(15)	10576	2021	6202	57
H(16)	10193	1526	4962	41
H(17A)	11005	778	737	26
H(17B)	10429	1149	1049	26
H(18A)	12545	1006	1570	31
H(18B)	12161	1279	578	31
H(20)	13275	1321	3188	37
H(21)	13290	1801	4550	48
H(22)	12092	2281	4426	51
H(23)	10860	2272	2929	54
H(24)	10829	1796	1570	45
H(25A)	8667	-808	4248	23
H(25B)	8204	-1085	5186	23
H(26A)	6710	-1165	4051	27
H(26B)	7046	-850	3174	27
H(28)	6678	-1726	3073	40
H(29)	7369	-2163	1782	52

H(30)	8933	-2043	822	41
H(31)	9734	-1476	1117	36
H(32)	9038	-1045	2380	30
H(33A)	6534	-232	2163	24
H(33B)	7055	70	1354	24
H(34A)	7695	-367	78	25
H(34B)	7525	-691	991	25
H(36)	6335	-224	-1333	34
H(37)	4762	-359	-2196	37
H(38)	3649	-777	-1301	42
H(39)	4101	-1034	532	50
H(40)	5687	-902	1360	46
H(41A)	10694	-543	2219	23
H(41B)	10073	-533	1013	23
H(42A)	11645	-502	-62	22
H(42B)	12284	-489	1127	22
H(44)	11554	-1093	-1002	31
H(45)	11604	-1734	-963	41
H(46)	11786	-2046	819	47
H(47)	12000	-1715	2549	46
H(48)	11928	-1072	2531	32
H(49)	6369	3144	6245	88
H(51)	3760	2550	6708	105
H(52)	4581	2330	8354	114
H(53)	6340	2308	8546	57
H(54)	7135	2828	7400	91
H(49')	6677	2999	6721	88
H(52')	3339	2836	5664	89
H(53')	4099	3327	4972	60
H(54')	5953	3383	5677	87

References

S1. Lee, C., Yang, W. & Parr, R. G. Local softness and chemical-reactivity in the molecules CO, SCN- and H_2CO. *J. Mol. Struct.* **40**, 305-313 (1988).

S2. Lee, C., Yang, W. & Parr, R. G. Development of the colle-salvetti correlation-energy formula into a functional of the electron density. *Phys. Rev. B* **37**, 785-789 (1988).

S3. Morrison, R. C., Yang, W., Parr, R. G. & Lee, C. Approximate density matrices and wigner distribution functions from density, kinetic energy density, and idempotency constraints. *Int. J. Quantum Chem.* **38**, 819-830 (1990).

S4. Yang, W., Parr, R. G. & Lee, C. Various functionals for the kinetic energy density of an atom or molecule. *Phys. Rev. A* **34**, 4586-4590 (1986).

S5. Hay, P. J. & Wadt, W. R. *Ab initio* effective core potentials for molecular calculations. Potentials for the transition metal atoms Sc to Hg. *J. Chem. Phys.* **82**, 270-283 (1985).

S6. Wadt, W. R. & Hay, P. J. *Ab initio* effective core potentials for molecular calculations. Potentials for main group elements Na to Bi. *J. Chem. Phys.* **82**, 284-298 (1985).

S7. Hay, P. J. & Wadt, W. R. *Ab initio* effective core potentials for molecular calculations. Potentials for K to Au including the outermost core orbitals. *J. Chem. Phys.* **82**, 299-310 (1985).

S8. Rassolov, V. A., Pople, J. A., Ratner, M. A. & Windus, T. L. 6-31G* basis set for atoms K through Zn. *J. Chem. Phys.* **109**, 1223-1229 (1998).

S9. Rassolov, V. A., Ratner, M. A., Pople, J. A., Redfern, P. C. & Curtiss, L. A. 6-31G* basis set for third-row atoms. *J. Comput. Chem.* **22**, 976-984 (2001).

S10. Frisch, M. *et al.* Gaussian, Inc., Wallingford, CT (2010).

S11. Kruger, T., Krebs, B. & Henkel, G. Nickel complexes containing sterically demanding thiolate ligands: $[Ni_8S(SC_4H_9)_9]^-$, a mixed-valence nickel sulfide thiolate cluster, and $[Ni_4(SC_3H7)_8]$, a homoleptic nickel thiolate. *Angew. Chem. Int. Ed.* **28,** 61-62 (1989).

S12. Kriege, M. & Henkel, G. $Ni_5(SET)_{10}$ and $Ni_4(SC_6H_{11})_8$, homoleptic nickel-thiolates with pentagonal-prismatic and cubane-like sulfur frameworks. *Z. Naturforsch. B* **42,** 1121-1128 (1987).

S13. Gaete, W., Ros, T., Solans, X. Font-Atltaba, M. & Brianso, J. L. Synthesis and crystal and molecular structure of *cyclo*-tetrakis[bis(μ-N-methylpiperidine-4-thiolato)nickel(II)]. *Inorg. Chem.* **23,** 39-43 (1984).

S14. Mahmoudkhani, A. H. & Langer, V. A novel oligomerization versus metal chelation: synthesis and crystal structure of pentakis[di-μ-(diisopropylaminoethanethiolato)nickel]. *Inorg. Chim. Acta* **294,** 83-86 (1999).

S15. Koo, B. K. Block, E. Kang, H. Liu, S. & Zubieta, J. Syntheis and structural characterization of *cyclo*-pentakis [bis(μ-trimethylsilylthiomethane)nickel(II)], $[Ni(SCH_2SiMe_3)_2]_5$, a pentametallic tiara structure. *Polyhedron* **7,** 1397-1399 (1988).

S16. Wark, T. A. & Stephan, D. W. Early metal thiolato species as metalloligands in the formation of early/late heterobimetallic complexes: syntheses and molecular structures of $Cp_2Ti(SMe)_2$, $Cp_2V(SMe)_2$, $(Cp_2Ti(\mu\text{-}SMe)_2)_2Ni$ and $(Ni(\mu\text{-}SMe)_2)_6$. *Organometallics* **8,** 2836-2843 (1989).

S17. Schulbert, K. & Mattes, R. Structure of 2 polynuclear nickel-thiolato-complexes $[(\mu\text{-}SMe)_2(Ni(MeNHCS_2))_2]$ and *cyclo*-$[(\mu\text{-}SMe)_2Ni]_6$. *Z. Naturforsch. B* **49,** 770-772 (1994).

S18. Woodward, P., Dahl, L. F., Abel, E. W. & Crosse, B. C. A new type of cyclic transition metal complex, $[Ni(SC_2H_5)_2]_6$. *J. Am. Chem. Soc.* **87,** 5251-5253 (1965).

S19. Miyamae, H. & Yamamura, T. Structure of the triclinic form of hexakis[di-μ-(ethanethiolato)nickel], and redetermination of its monoclinic form. *Acta Crystallogr. C* **44,** 606-609 (1988).

S20. Feld, H. *et al.* High mass resolution plasma desorption and secondary ion mass-spectrometry of neutral nickel thiolate complexes- crystal-structure of $Ni_6(SC_3H_7)_{12}$. *Z. Naturforsch. B* **47,** 929-936 (1992).

S21. Xiao, H. L., Jian, F. F. & Zhang, K. J. Synthesis and structure analysis of α and β forms of [12] metallacrown-6 nickel(II) complex: $[Ni_6(SCH_2CH_2CH_3)_{12}]$. *Bull. Korean Chem. Soc.* **30,** 846-848 (2009).

S22. Gould, R. O. & Harding, M. M. Nickel and palladium complexes of 1-hydroxyethane-2-thiol and analogues. Part I. Crystal structure of cyclohexakis[bis-(μ-1-hydroxyethane-2-thiolato)-nickel(II)]. *J. Chem. Soc. A* 875-881 (1970).

S23. Jian, F.-F., Jiao, K., Li, Y., Zhao, P.-S. & Lu, L.-D. $[Ni_6(SCH_2CH_2OH)_{12}]$: A double crown [12]metallacrown-6 nickel(II) cluster. *Angew. Chem. Int. Ed.* **42,** 5722-5724 (2003).

S24. Sletten, J. & Kovacs, J. A. The structure of a toroidal, neutral, homoleptic Ni(II) complex with a chelate dithiolate ligand, $[Ni_6(SCH_2CH_2CH_2S)_6]$. *Acta Chem. Scand.* **48,** 929-932 (1994).

S25. Tan, C. *et al.* In situ synthesis of nickel tiara-like clusters with two different thiolate bridges. *Dalton Trans.* **41,** 8472-8476 (2012).

S26. Zhang, C., Takada, S., Kolzer, M., Matsumoto, T. & Tatsumi, K. Nickel(II) thiolate complexes with a flexible *cyclo*-$\{Ni_{10}S_{20}\}$ framework. *Angew. Chem. Int. Ed.* **45,** 3768-3772 (2006)

S27. Capdevila, M. *et al.* Preparation and X-ray crystal structure of $[Ni_6\{\mu\text{-}S(CH_2)_3N(CH_3)_2\}_{12}]$, a cyclic hexameric homothiolate of nickel. *Polyhedron* **8,** 1253-1259 (1989).

S28. Barrera, H., Bayon, J. C., Suades, J., Germain, C. & Declerq, J. P. Metal complexes of mercaptoamines-III. Different behaviour of β- and γ-(N,N-dimethyl)-mercaptoamines. *Polyhedron* **3,** 969-975 (1984).

S29. Angamuthu, R. & Bouwman, E. Reduction of protons assisted by a hexanuclear nickel thiolate metallacrown: protonation and electrocatalytic dihydrogen evolution. *Phys. Chem. Chem. Phys.* **11,** 5578-5583 (2009).

S30. Cai J.-H. & Kang B.-S. Synthesis and structure of hexakis [di-μ-(4-chloro-brnzylthio) nickel] tritetrachloromethane solvate, $[Ni(SCH_2C_6H_4Cl\text{-}p)_2]_6{\cdot}3CCl_4$. *Jiegou Huaxue (Chinese J. Struct. Chem.)* **12,** 397-400(1993).

S31. Mahmoudkhani, A. H. & Langer, V. Structural investigation of nickel thiolates: a cyclic hexanuclear nickel thiolate with highly distorted NiS_4 units. *Polyhedron* **18,** 3407-3410 (1999).

S32. Zhu, M., Zhou, S., Yao, C., Liao, L. & Wu, Z. Reduction-resistant and reduction-catalytic double-crown nickel nanoclusters. *Nanoscale* **6,** 14195-14199 (2014).

S33. Kagalwala, H. N. *et al.* Photocatalytic hydrogen generation system using a nickel-thiolate hexameric cluster. *Inorg. Chem.* **52,** 9094-9101 (2013).

S34. Dance, I. G., Scudder, M. L. & Secomb, R. *c*-$Ni_8(SCH_2COOEt)_{16}$, a receptive octagonal toroid. *Inorg. Chem.* **24,** 1201-1208 (1985).

S35. Ivanov, S. A., Kozee, M. A., Alex M, W., Agarwal, S. & Dahl, L. F. *Cyclo*-$[Ni(\mu_2\text{-}SPh)_2]_9$ and *cyclo*-$[Ni(\mu_2\text{-}SPh)_2]_{11}$: new oligomeric types of toroidal nickel(II) thiolates containing geometrically unprecedented 9- and 11-membered ring systems. *J. Chem. Soc., Dalton Trans.* 4105-4115 (2002).

S36. Zhang, C. *et al.* Dodecanuclear-ellipse and decanuclear-wheel nickel(II) thiolato clusters with efficient femtosecond nonlinear absorption. *Angew. Chem. Int. Ed.* **49,** 4209-4212 (2010).

S37. Stash, A. I., Perepelkova, T. I., Noskov, Y. G., Buslaeva, T. M. & Romm, I. P. Palladium clusters $Pd_4(SEt)_4(OAc)_4$ and $Pd_6(SEt)_{12}$: structure and properties. *Russ. J. Coord. Chem.* **27,** 585-590 (2001).

S38. Kunchur, N.G. The crystal structure of palladium n-propyl mercaptide. *Acta Crystallogr. B* **24,** 1623-1633 (1968).

S39. Higgins, J. D. & William Suggs, J. Preparation, structure and spectroscopic studies of the palladium mercaptides $Pd_8(S\text{-}nPr)_{16}$ and $Pd_6(S\text{-}nPr)_{12}$. *Inorg. Chim. Acta* **145,** 247-252 (1988).

S40. Stash, A. I. *et al.* Palladium clusters $Pd_4(SR)_4(OAc)_4$ and $Pd_6(SR)_{12}$ (R = Bu, Ph): Structure and properties. *Russ. J. Coord. Chem.* **35,** 136-141 (2009).

S41. Ananikov, V. P. *et al.* Catalytic adaptive recognition of thiol (SH) and selenol (SeH) groups toward synthesis of functionalized vinyl monomers. *J. Am. Chem. Soc.* **134,** 6637-6649 (2012).

S42. Yang, Z., Smetana, A. B., Sorensen, C. M. & Klabunde, K. J. Synthesis and characterization of a new tiara Pd(II) thiolate complex, $[Pd(SC_{12}H_{25})_2]_6$, and its solution-phase thermolysis to prepare nearly monodisperse palladium sulfide nanoparticles. *Inorg. Chem.* **46,** 2427-2431 (2007).

S43. Schneider, I., Horner, M. & Olendzki, R. N. Structure of cyclo-hexakis[bis-μ-(methoxycarbonylmethylthiolato)-palladium(II)], $[Pd(SCH_2COOCH_3)_2]_6$. *Acta Crystallogr. C* **49,** 2091-2093 (1993).

S44. Yamashina, Y., Kataoka, Y. & Ura, Y. Tiara-like octanuclear palladium(II) and platinum(II) thiolates and their inclusion

complexes with dihalo- or iodoalkanes. *Inorg. Chem.* **53,** 3558-3567 (2014).
